# An overview of the postcranial osteology of caecilians (Gymnophiona, Lissamphibia)

**DOI:** 10.1002/ar.70000

**Published:** 2025-06-06

**Authors:** Rodolfo Otávio Santos, Mark Wilkinson, Hussam Zaher

**Affiliations:** ^1^ Instituto de Biociências Universidade de São Paulo São Paulo São Paulo Brazil; ^2^ Serviço de Vertebrados Museu de Zoologia da Universidade de São Paulo São Paulo São Paulo Brazil; ^3^ Herpetology The Natural History Museum London UK

**Keywords:** axial skeleton, comparative anatomy, herpetology, vertebral column

## Abstract

Caecilians comprise a relatively small (~220 species) group (Gymnophiona) of snake‐like or worm‐like, mostly tropical amphibians. Most adult caecilians are fossorial, although some species may live in aquatic or semi‐aquatic environments, either as larvae or adults. Caecilians exhibit numerous morphological features traditionally interpreted as adaptations to their specialized ecologies, such as a compact and well‐ossified skull and an elongated body lacking both girdles and limbs. Caecilian vertebrae differ substantially from those of other amphibians in having amphicoelous centra, well‐developed basapophyseal processes, pronounced posterosagittal processes and hypapophyseal keels, and low and flat neural arches. However, caecilian postcranial osteology has received little attention, and the vast majority of the species remain unstudied. Consequently, the variation in the vertebral morphology among caecilians is still unknown or poorly documented. Inconsistencies in the anatomical terminology used by different authors are potentially confusing and may hamper understanding of homologies. Here we present an overview of caecilian postcranial osteology, define the main structural features, including many not previously described, and propose a standardized nomenclature.

## 1 INTRODUCTION

Caecilians (Gymnophiona) are a relatively small clade (~220 recent species) of amphibians with a distinct morphology characterized, among other features, by a complete lack of limbs and girdles (Wagler, 1830), such that the postcranial skeleton of modern caecilians comprises only vertebrae and ribs. Caecilians are often referred to as the least known group of tetrapods (e.g., Wake, 2003; Wilkinson, 2012), and this is particularly true of their postcranial osteology, which, despite intermittent study over more than 200 years, remains incompletely documented and understood.

The study of caecilian postcranial skeleton commenced at the beginning of the 19th century, shortly after the recognition of caecilians as a distinct group and the earliest scientific descriptions of species. Most of the early works were based on very limited materials and primarily concerned with the comparison of caecilians to other taxa and what that might reveal about the positioning of Gymnophiona within the Vertebrata (e.g., Müller, 1831; Wiedersheim, 1879). These previous studies of caecilian vertebral columns have varied in scope (see below) and, in combination, represent a substantial corpus on caecilian postcranial osteology.

However, knowledge of the diversity of vertebral structure in caecilians remains incomplete, and there are many potentially confusing differences in the descriptive terminologies that previous authors have employed. Here we provide a new characterization of the anatomy of caecilian vertebrae and ribs. We describe the general patterns and morphological variation present in Gymnophiona based on investigations of representatives of all 10 currently recognized extant families (Kamei et al., 2012; Wilkinson et al., 2011). We seek to clarify and standardize existing terminology based on the recognition of putative homologies and thereby improve congruence with anatomical nomenclature used for other vertebrate groups. We introduce several new or replacement terms for previously unreported, unnamed, or confusingly or misleadingly named features. In doing so, we aim to establish a solid framework for further research in comparative anatomy and related disciplines. In the following sections, we provide a concise yet comprehensive historical overview, a detailed morphological characterization of the caecilian vertebral column elements, and a discussion on anatomical terminology and comparative anatomy.

## 2 HISTORICAL BACKGROUND

In this section, we highlight some noteworthy contributions to the development of knowledge of the caecilian vertebral column and ribs, mostly excluding studies that reported on a single species. A more comprehensive listing of previous works and their scope is given in Table 1. We use current taxonomic names rather than those used by the original authors, while acknowledging that some uncertainty may exist regarding how past names align with current taxonomic concepts. Similarly, we use our preferred anatomical terminology when this differs from the names of structures used by previous authors.

**TABLE 1:** List of previous works dealing with caecilian postcranial osteology and their respective scopes.

Work	Scope
Schneider (1801)	1 genus (*Caecilia*)
Wagler (1830)	3 genera (*Caecilia*, *Ichthyophis*, and *Siphonops*)
Müller (1831)	2 species (*Caecilia gracilis* and *Ichthyophis glutinosus*)
Stannius (1856)	3 genera (*Caecilia*, *Ichthyophis*, and *Siphonops*)
Gegenbaur (1862)	1 species (*Caecilia gracilis*)
Wiedersheim (1879)	5 species (*C. gracilis*, *Chthonerpeton indistinctum*, *Hypogeophis rostratus*, *I. glutinosus*, and *Siphonops annulatus*)
Frič (1883)	1 species (*I. glutinosus*)
Peter (1894)	6 species (*C. indistinctum*, *H. rostratus*, *I. glutinosus*, *Schistometopum thomense*, *S. annulatus*, and *Uraeotyphlus oxyurus*)
Göppert (1896)	1 species (*I. glutinosus*)
Marcus & Blume (1926)	2 species (*Grandisonia alternans* and *H. rostratus*)
Bolk et al. (1936)	2 species (*S. thomense* and *H. rostratus*)
Lawson (1963)	1 species (*H. rostratus*)
Taylor (1968)	20 genera (*Ichthyophis*, “*Caudacaecilia*”, *Epicrionops*, *Typhlonectes*, *Potomotyphlus*, *Nectocaecilia*, *Chthonerpeton*, *Caecilia*, *Dermophis*, *Microcaecilia*, “*Pseudosiphonops*,” *Oscaecilia*, *Schistometopum*, *Herpele*, *Uraeotyphlus*, *Geotrypetes*, *Gegeneophis*, *Indotyphlus*, “*Grandisonia*,” and *Hypogeophis*)
Estes & Wake (1972)	7 species (*Apodops pricei*, *Dermophis mexicanus*, *Geotrypetes seraphini*, *Gymnopis multiplicata*, *H. rostratus*, *S. thomense*, and *Typhlonectes compressicauda*)
Taylor (1977)	24 species (*Caecilia albiventris*, *Caecilia degenerata*, *Caecilia disossea*, *Caecilia occidentalis*, *Caecilia orientalis*, *Gegeneophis ramaswamii*, *G. seraphini*, *G. alternans*, *G. multiplicata*, *D. mexicanus*, *H. rostratus*, *Ichthyophis beddomei*, *Ichthyophis kohtaoensis*, *Ichthyophis larutensis*, *Ichthyophis mindanaoensis*, *Ichthyophis nigroflavus*, *Oscaecilia bassleri*, *Oscaecilia ochrocephala*, *Schistometopum gregorii*, *Scolecomorphus kirkii*, *S. annulatus*, *Siphonops paulensis*, *Typhlonectes natans*, and *U. oxyurus*)
Naylor & Nussbaum (1980)	6 species (*Epicrionops petersi*, *H. rostratus*, *I. glutinosus*, *Scolecomorphus vittatus*, *Typhlonectes* sp., and *Uraeotyphlus narayani*)
Wake (1980)	3 species (*D. mexicanus*, *I. glutinosus*, and *T. compressicauda*)
Wake (1986)	1 species (*ldiocranium russeli*)
Wake (1987)	2 species (*G. seraphini* and *Sylvacaecilia grandisonae*)
Azpelicueta et al. (1987)	1 species (*C. indistinctum*)
Renous & Gasc (1989)	32 genera (“*Afrocaecilia*”, *Boulengerula*, *Brasilotyphlus*, *Caecilia*, *Chthonerpeton*, “*Caudacaecilia*”, *Dermophis*, *Epicrionops*, *Gegeneophis*, *Geotrypetes*, *Grandisonia*, *Gymnopis*, *Herpele*, *Hypogeophis*, *Ichthyophis*, *Idiocranium*, *Indotyphlus*, *Luetkenotyphlus*, *Microcaecilia*, *Mimosiphonops*, *Nectocaecilia*, *Oscaecilia*, “*Parvicaecilia*,” *Praslinia*, *Potomotyphlus*, “*Pseudosiphonops*,” *Rhinatrema*, *Schistometopum*, *Scolecomorphus*, *Siphonops*, *Typhlonectes*, and *Uraeotyphlus*)
Rage (1991)	1 indeterminate fossil taxon
Wilkinson (1992)	1 species (*Herpele squalostoma*)
Evans et al. (1996)	1 indeterminate fossil taxon
Wilkinson & Nussbaum (1997, 1999)	6 species (*Atretochoana eiselti*, *C. indistinctum*, *Nectocaecilia petersii*, *Potomotyphlus kaupii*, *T. compressicauda*, and *T. natans*)
Wake et al. (1999)	1 species (presumably *D. mexicanus*)
Evans & Sigogneau‐Russell (2001)	1 species (*Rubricaecilia monbaroni*)
Gayet et al. (2001)	1 indeterminate fossil taxon
Wake (2003)	16 species (*Boulengerula taitana*, *Caecilia occidentalis*, *D. mexicanus*, *Epicrionops bicolor*, *G. seraphini*, *G. multiplicata*, *H. rostratus*, *Ichthyophis* sp., *Idiocranium russelli*, *O. ochrocephala*, *S. thomense*, *S. kirkii*, *Scolecomorphus uluguruensis*, *S. grandisonae*, *T. natans*, *U. narayani*)
Jenkins et al. (2007)	1 species (*Eocaecilia micropodia*)
Wilkinson et al. (2021)	1 species (*Amazops amazops*)
Lowie et al. (2022b)	28 species (*A. eiselti*, *Boulengerula boulengeri*, *Boulengerula fischeri*, *B. taitana*, *Caecilia museugoeldi*, *Caecilia tentaculata*, *D. mexicanus*, *E. bicolor*, *G. ramaswamii*, *G. seraphini*, *G. alternans*, *H. squalostoma*, *H. rostratus*, *Ichthyophis bombayensis*, *I. kohtaoensis*, *Microcaecilia unicolor*, *Mimosiphonops vermiculatus*, *P. kaupii*, *Rhinatrema bivittatum*, *S. gregorii*, *S. thomense*, *S. kirkii*, *S. uluguruensis*, *S. annulatus*, *S. grandisonae*, *T. compressicauda*, *T. natans*, and *U. oxyurus*)
Lowie et al. (2022a)	13 species (*B. fischeri*, *B. taitanus*, *C. museugoeldi*, *C. tentaculata*, *D. mexicanus*, *G. seraphini*, *H. squalostoma*, *H. rostratus*, *I. kohtaoensis*, *R. bivittatum*, *S. thomense*, *S. annulatus*, and *T. compressicauda*)
Palakkool et al. (2022)	1 species (*Gegeneophis carnosus*)
Santos et al. (2024)	4 species (*Ymboirana acrux*, *C. indistinctum*, *P. kaupii*, and *N. petersii*)

Seemingly overlooked by all subsequent authors, the first contribution to caecilian vertebral column morphology that we have found is Schneider's (1801) brief but accurate description of vertebrae and ribs of an indeterminate species. Schneider (1801) noted some important features of caecilian trunk vertebrae, including amphicoelous centra with ligamentous intervertebral connections, paired anteriorly directed basipophyseal processes, unpaired median posterosagittal processes, poorly developed neural spines, and short bicapitate ribs. Wagler (1830) also reported that the ribs are short and noted the presence of cartilage between adjacent centra, that caecilians lack a sternum, and that there is no trace of girdles. Attributed to *Ichthyophis glutinosus* (Linnaeus), Müller (1831, figure 16 in plate XXI) provided the first illustrations of components of the vertebral column of any caecilian of which we are aware. He considered caecilian vertebrae to be very similar to those of the salamander *Proteus* Laurenti, 1768, and reported that vertebral size may vary with position in the column.

**FIGURE 1:**
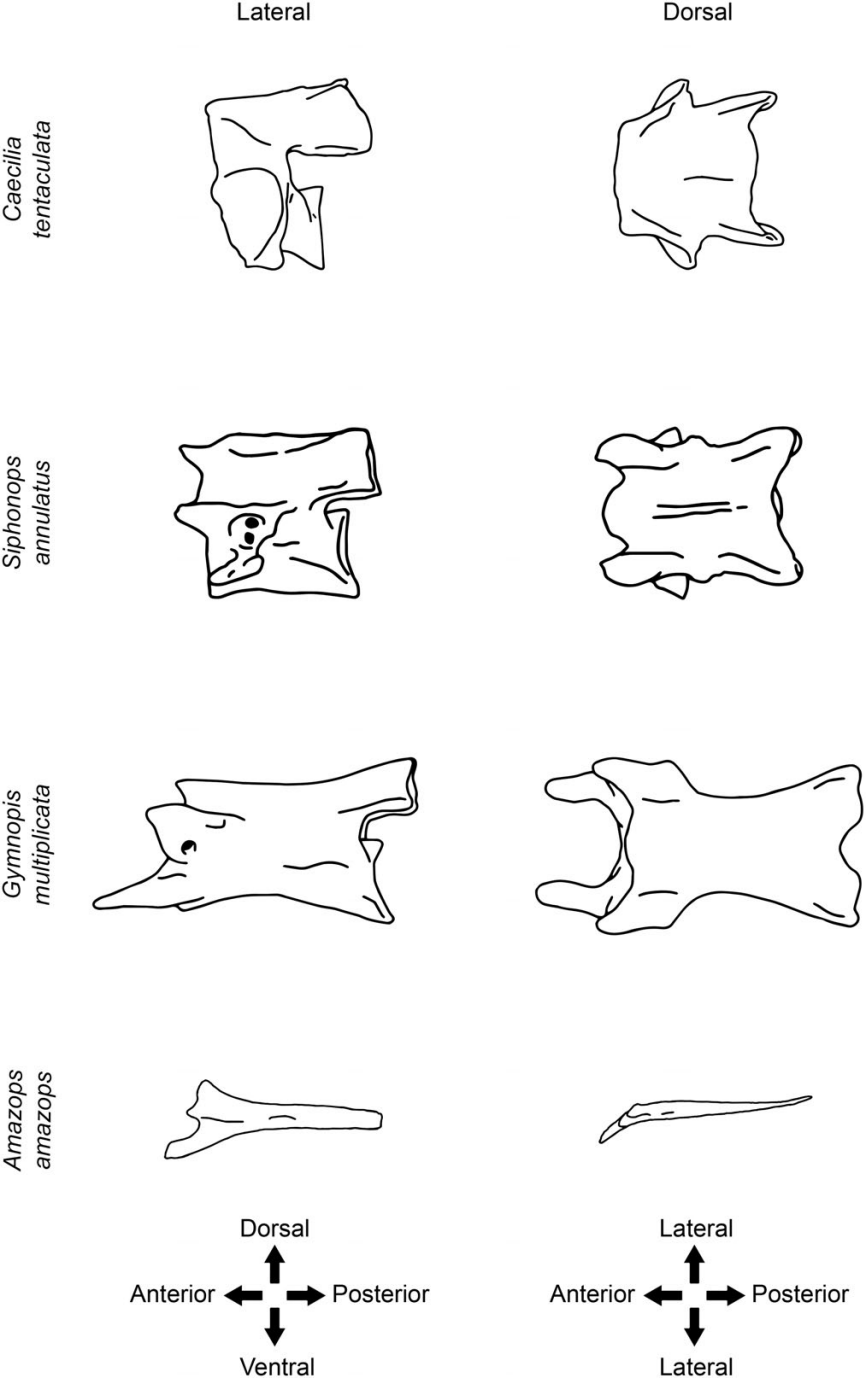
Schematic drawings of caecilian vertebrae and rib in lateral and dorsal views, highlighting the anatomical orientations followed in the present work. Atlas of *Caecilia tentaculata*, Second vertebra of *Siphonops annulatus*, Trunk vertebra of *Gymnopis multiplicata*, Rib of *Amazops amazops*. Figures not in scale.

Gegenbaur (1862) illustrated (Figure 1, plate I) a longitudinal section through vertebral centra of *Caecilia gracilis* Shaw, 1802 and discussed caecilian vertebral structure from the perspective of development and in relation to the notochord. He confirmed the presence of intervertebral ligaments and cartilages between the centra. In his monograph, Die Anatomie der Gymnophionen, Wiedersheim's (1879) surveyed diverse aspects of caecilian anatomy and inferred a close relationship with the salamanders. Based on examination of four species, the typhlonectid *Chthonerpeton indistinctum* (Reinhardt & Lütken, 1862), grandisoniid *Hypogeophis rostratus* (Cuvier, 1829), caeciliid *C. gracilis*, and ichthyophiid *I. glutinosus*, Wiedersheim provided more detailed descriptions of caecilian vertebrae and ribs than previous workers and noted some differences between caecilian taxa. Among his general findings are the presence of bicapitate ribs that articulate with dorsal and ventral transverse processes (as in salamanders), foramina for vertebral arteries, zygapophyses with cartilage‐covered articular facets, and the distinctive posteroventral arrowhead‐shaped posterosagittal process that extends between the basapophyseal processes of the next posterior vertebra.

Frič (1883) described several fossil Aïstopoda, noted some similarities with the vertebrae of caecilians, and provided drawings of a trunk vertebra of *I. glutinosus*. Peter (1894) provided the first scientific work devoted exclusively to the postcranial anatomy of caecilians and the first that is not mostly limited to comparative description. It is impressive both in the accounts of anatomy and in the generally plausible and insightful interpretation of structure in terms of natural history, function, and evolution. It included illustrations and the then most detailed descriptions of vertebrae and ribs of several taxa, consideration of intraspecific and ontogenetic variation, and made extensive comparisons to salamanders, to which he concluded caecilians were closely related. He also noted that the similarities between the vertebrae of caecilians and aïstopods recognized by Frič (1883) were only superficial and possibly related to similar lifestyles. Göppert (1896) published a work focused on the morphology of the amphibian ribs and discussed putative homology with the ventral ribs of fishes. He also appears to provide the first, hitherto overlooked, report of haemal arches in a caecilian (in the tail of *I. glutinosus*).

Abel (1919) used the morphology of the vertebral column in his proposed classification of vertebrates and included caecilians in the group of tetrapods with pseudocentrous vertebrae (i.e., formed from the fusion of dorsal and ventral arcualia). Marcus & Blume (1926) provided an extensive description of the vertebrae and ribs of young and adult representatives of *Hypogeophis alternans* Stejnegar, 1893, and *H. rostratus*. In his treatise on the evolution of the vertebral column, Gadow (1933) proposed a standardized terminology for the structures present in the vertebrae of tetrapods, including some features exclusively known in caecilians. He also employed the structure of the vertebrae as the basis for his own proposal of tetrapod classification, in which caecilians and salamanders were included in the grade of Lepospondylous amphibia by virtue of their pseudocentrous vertebrae. Bolk et al. (1936) included a brief description of caecilian vertebrae in their book on the anatomy of vertebrates.

The number of vertebrae, determined either from osteological preparations and more commonly from radiographs, has been employed in caecilian systematics (e.g., Parker, 1958). Taylor (1968) discussed the correlation between the number of vertebrae and primary annuli, and provided vertebral counts and x‐ray images for several species. Estes & Wake (1972) described a fossil caecilian based on an isolated vertebra and compared it with material from extant species. Taylor (1977) provided a broadly comparative study of the anteriormost (“cervical”) vertebrae of caecilians, including many illustrations revealing differences considered to be (p. 220) “taxonomically useful for potential distinction of genera and/or species in the several families of caecilians”. Naylor & Nussbaum (1980) described the trunk muscles of caecilians in relation to the vertebral column. With comparisons to a few other caecilian species, Wake (1980) focused on the postcranial anatomy of *Dermophis mexicanus* (Dumeril & Bibron, 1841) using a morphometric approach and also described morphological variation related to ontogeny. Wake (1986) described the anatomy of *ldiocranium russeli* Parker, 1936, including aspects of its postcranial osteology, noting that the posterior elements of the column exhibit an incomplete development, interpreted as a result of miniaturization through heterochrony.

The numbers of nuchal and postcloacal vertebrae have also sometimes been considered in systematic works (e.g., Lescure et al., 1986; Nussbaum & Wilkinson, 1989; Wilkinson, 1989) with the latter known to vary artefactually with preservation (Nussbaum, 1988). Renous & Gasc (1989) used data on total length and vertebral counts from Taylor (1968) and employed a morphometric approach to propose that the variation in the vertebral proportions of caecilians may be correlated with their locomotor behavior. Wilkinson & Nussbaum (1997) provided detailed descriptions of the anatomy of representatives of the family Typhlonectidae, including some aspects of their postcranial osteology and Wilkinson & Nussbaum's (1999) phylogenetic hypothesis for this family was based on morphological data including 17 new characters drawn from postcranial osteology; as yet, the only use of caecilian vertebral characters (other than just vertebral count) in phylogenetic inference including only living forms. Wake et al. (1999) reported the discovery of an isolated caecilian vertebra from a Quaternary site and interpreted it as a trunk vertebra of *D. mexicanus*.

Wake (2003) presented a review of caecilian osteology including vertebrae and ribs. In their monograph on the anatomy of *Eocaecilia micropodia*, Jenkins & Walsh, 1993, Jenkins et al. (2007) described features of its postcranial osteology and provided anatomical comparisons between it and extant caecilians. Santos et al. (2020) reviewed the fossil record of caecilians, which is primarily composed of isolated vertebrae. Using a morphometric approach applied to a representative sample (24 species), Lowie et al. (2022b) described variation and disparity in the shape of caecilian vertebrae along the column based on comparison of vertebrae from five putatively corresponding positions (following Wake, 1980). Lowie et al. (2022a) proposed a correlation between vertebral morphology and the forces generated during active burrowing. Santos et al. (2024) described a new species of aquatic caecilian based on fossil material (including vertebrae and ribs) and compared the postcranial anatomy of the newly described taxon with other extant typhlonectids.

## 3 MATERIALS AND METHODS

### 3.1 Examined specimens

Our morphological study is based on observations of specimens assigned to 84 living caecilian taxa, encompassing all currently recognized caecilian families. A complete list of the studied specimens is available in Supporting Information S1. Observations were made from computed tomography scans (CT‐scans), radiographs, dried skeletons, and cleared and stained specimens. Please refer to the Supporting Information S1 for the detailed parameters used in the CT‐scan procedures of the figured specimens. Images of the vertebrae and ribs were made using VG Studio MAX 3.4.5 64 bits (Volume Graphics, http://www.volumegraphics.com).

### 3.2 Terminology

Unsurprisingly, given the long but intermittent history of caecilian postcranial osteology, different terms have sometimes been used by different authors to describe the same structures of the vertebrae or ribs, and the same terms have sometimes been applied to different, non‐homologous structures within caecilians or other vertebrates. A summary of variation in anatomical terminology is given in Table 2, and an illustrated glossary of anatomical terms is provided at the end of this work. The anatomical orientations of the vertebrae and ribs are depicted in Figure 1. All abbreviations are listed in Appendix A.

**TABLE 2:** Different terminologies applied to the caecilian postcranial osteology.

This work	Basapophyseal process	Parapophysis	Hypapophyseal keel	Posterosagittal process	Interglenoid tubercle	Neural spine
Wiedersheim (1879)^a^	Processus transversus inferiores	—	Basal keel	Processus spinosus posterior	Processus odontoideum	Processus spinosus
Peter (1894)^a^	Processus inferoanteriores	Parapophysis	Median crest	Processus inferoposterior	Processus odontoides	Spinous process
Marcus & Blume (1926)^a^	Anterior inferior process	Parapophysis	Inferior median crest	Inferoposterior process	Odontoid process	Neural spine
Lawson (1963)	—	Ventral transverse process	Ventral ridge	Ventral spine	—	Neural spine
Estes & Wake (1972)	Parapophysis	—	Ventral spine	—	—	Neural spine
Taylor (1977)	Ventral process	—	Ventral ridge	—	—	Dorsal ridge
Naylor & Nussbaum (1980)	Basapophysis		Subcentral keel	—	—	—
Wake (1980)	Parapophysis	Parapophysis	Ventral keel	—	Tuberculum interglenoideum	Nuchal keel
Wake (1986)	—	Parapophysis	Ventral keel	—	—	Neural arch keel
Wake (1987)	Hypapophyses (hyperapophyses?)	—	—	—	—	Nuchal (dorsal) keel
Azpelicueta et al. (1987)^a^	—	Parapophysis	Ventral keel	—	—	—
Rage (1991)	Parapophyseal process	Parapophysis	—	—	—	—
Wilkinson (1992)	Parasphenes	—	Ventral keel	Zygosphene	—	—
Evans et al. (1996)	Parapophysis	Rib facet	Midventral keel	—	—	—
Wilkinson & Nussbaum (1997, 1999)	Parasphenes	Parapophysis	—	Hypapophysis	—	Nuchal crest
Wake et al. (1999)	Parapophysis	Articular facet	Ventral keel	—	—	Keel
Evans & Sigogneau‐Russell (2001)	Basapophysis	Parapophysis	Midventral keel	—	Tuberculum interglenoideum	Midline ridge
Gayet et al. (2001)	Anterior process	—	Sagital keel	—	—	—
Wake (2003)	Parapophysis	—	Ventral keel	—	Tuberculum interglenoideum	Nuchal keel
Jenkins et al. (2007)	Parapophyseal processes	Parapophysis	Median keel	—	Interglenoid tubercle	Nuchal keel (spinous process)
Wilkinson et al. (2021)	Parasphenes	—	—	Hyposphene	—	—
Lowie et al. (2022b)	Basapophyseal processes	—	Hypapophyseal keel	Hypapophysis	—	—
Palakkool et al. (2022)	Parasphene (basapophysis)	Parapophyseal facet	Ventral (hypapophyseal) keel	Hypapophysis	—	Nuchal ridge

^a^ Works originally published in languages other than English.

In choosing terminology, we prefer names that are correlated with assumed function, position, or association with other structures and to be as consistent as possible with terminology applied in other associated areas (e.g., myology) and to other taxa. We have attempted to avoid potential ambiguities by providing definitions of each anatomical term and, where relevant, considering potential homologies between the caecilians and other vertebrates, particularly other amphibians. Where sensible, we have attempted to promote stability by conserving existing names. Some further elaboration of our general and some specific reasoning regarding the adopted terminology is provided in the discussion.

### 3.3 General remarks on caecilian postcranial osteology

The vertebral column of modern caecilians comprises an ordered series of serially homologous fundamental units, each comprising a vertebra with or without a pair of ribs. Its linear form lends itself to a natural anterior‐to‐posterior numbering of individual units, which can be helpful in identifying individual vertebrae and identifying which vertebrae are associated with other anatomical features, such as the nuchal collars or vent. Caecilians show variation in the number of vertebrae from as few as 67 to as many as 306 (Lowie et al. 2022a). Thus, the same anterior‐to‐posterior numbering of an individual unit in different individuals need not (and usually does not) imply a specific inter‐individual homology of those elements. A fractional indexing (e.g., *x*/*n*, where *x* is the number of the element and *n* is the total number) or the equivalent percentage indexing system as used by Wake (1980) and Lowie et al. (2022b) might enhance homology or at least comparability of elements with similar indices. Where the focus is on the posteriormost vertebrae, a posterior‐to‐anterior indexing may prove helpful, but to avoid confusion with the anterior–posterior indices, we have found it useful to arbitrarily label the posteriormost vertebra as zero and give successive negative numbers to those preceding it. Composite structures seemingly arising from the fusion of adjacent vertebrae are not uncommon in caecilians. In such cases, for the purposes of counting numbers of vertebrae or using their position in the series as an index, we suggest counting each presumed fundamental vertebral unit separately and noting, if needed, those which are conjoined.

**FIGURE 2:**
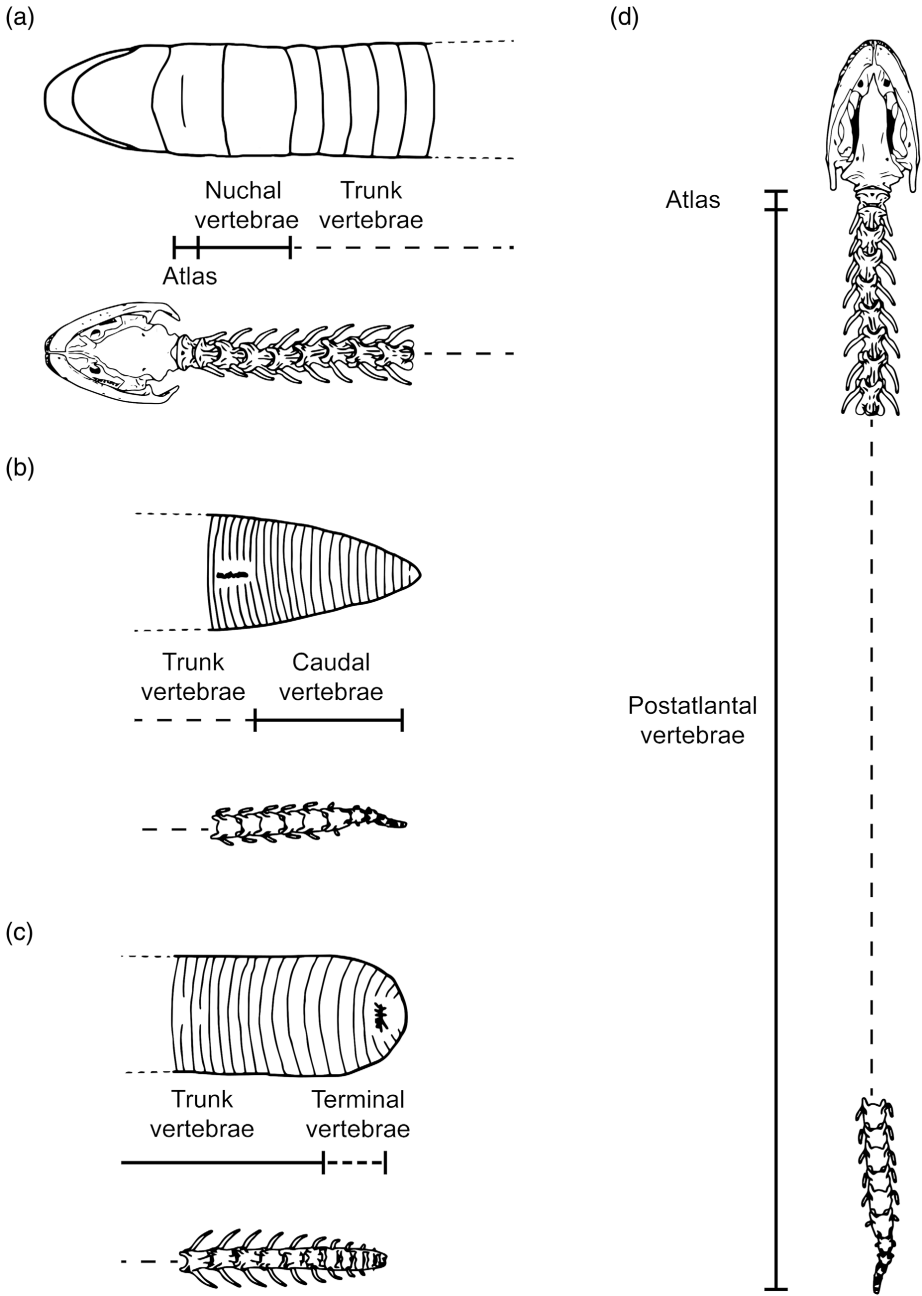
Schematic drawings representing the different possibilities of subdivision in the vertebral column in caecilians. Dashed lines indicate regions not delimited by any fixed external landmark. Figures not to scale.

Caecilians lack any trace of limbs or girdles (Wagler, 1830) and thus have no clear regional subdivisions of the vertebral column based on the regional boundaries that the positions of girdles provide in most other vertebrates (Peter, 1894). Some regional subdivision is nonetheless possible, and we adopt the following scheme (summarized in Figure 2). The atlas is the first (anteriormost vertebra); the remaining vertebrae are postatlantal. Trunk vertebrae are postatlantal vertebrae that are not caudal vertebrae. Caudal vertebrae are vertebrae posterior to the vent in caecilians that have a true tail as defined by Nussbaum & Wilkinson (1989), that is, a postcloacal region with both vertebrae and annular grooves. In the absence of a true tail, we refer to the posteriormost trunk vertebrae as terminal. Taylor (1977: 220) proposed using a functional criterion for distinguishing the four or five anteriormost vertebrae of caecilians as “cervicals” because they function together in critical pivoting movements of the cranium. Preferring a definition based on position (functional differences notwithstanding), we instead follow Lescure et al. (1986) in denoting the three to six anteriormost postatlantal vertebrae that lie completely or partially within the nuchal collars (i.e., anterior to the third nuchal groove) as nuchal vertebrae.

The illustrated descriptive accounts that follow are intended to summarize the major features and main forms of variation encountered in caecilian vertebrae and ribs.

## 4 RESULTS

### 4.1 Atlas

**FIGURE 3:**
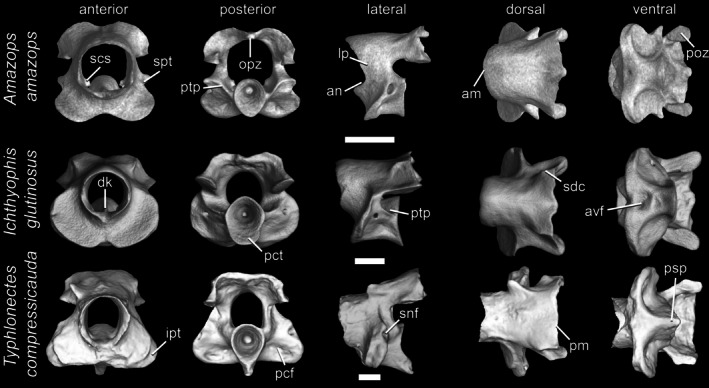
Atlases of *Amazops amazops*, *Ichthyophis glutinosus*, and *Typhlonectes compressicauda*, in anterior, posterior, lateral, dorsal, and ventral views. Scale bars = 1 mm. am, anterior margin of the neural arch; an, anterior notch; avf, atlantal ventral fossa; dk, dorsal keel of the vertebral centrum; ipt, infracotylar protuberance; lp, lateral pedicles; opz, opisthozygosphene; pcf, postcotylar fossa; pct, posterior cotyle; pm, posterior margin of the neural arch; poz, postzygapophysis; psp, posterosagittal process; ptp, posterior pedicles; scs, spinal cord supports; sdc, secondary dorsal crests; snf, spinal nerve foramen; spt, supracotylar protuberance.

**FIGURE 4:**
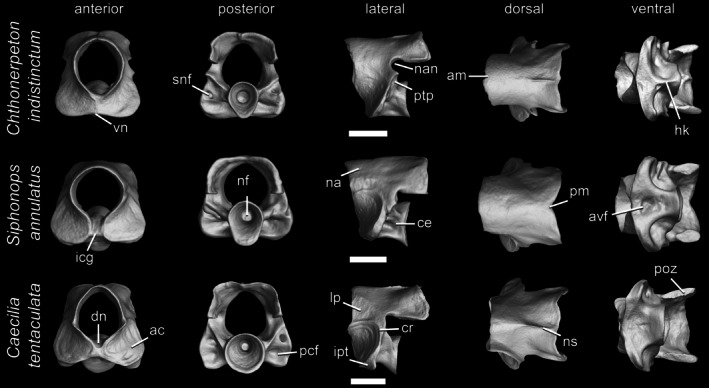
Atlases of *Chthonerpeton indistinctum*, *Siphonops annulatus*, and *Caecilia tentaculata*, in anterior, posterior, lateral, dorsal, and ventral views. Scale bars = 1 mm. ac, atlantal cotyles; am, anterior margin of the neural arch; avf, atlantal ventral fossa; ce, centrum; cr, cotylar rim; dn, dorsal notch; hk, hypapophyseal keel; icg, intercotylar gap; ipt, infracotylar protuberance; lp, lateral pedicles; na, neural arch; nan, neural arch notch; nf, notochordal fossa; ns, neural spine; pcf, postcotylar fossa; pm, posterior margin of the neural arch; poz, postzygapophysis; ptp, posterior pedicles; snf, spinal nerve foramen; vn, ventral notch.

The atlas is the first (i.e., anteriormost) and most distinctively individual vertebra (Figures 3 and 4). Its paired atlantal cotyles articulate anteriorly with the occipital condyles of the back of the skull, and its postzygapophyses articulate posteriorly with the prezygapophysis, and in some cases its posterosagittal process also articulates with the basapophyseal processes of the second vertebra. Unlike salamanders and albanerpetids, modern caecilians lack an interglenoid tubercle between the atlantal cotyles (McGowan, 1998; Peter, 1894; Wake, 1970). The atlas can be subdivided into three distinct main parts or regions: atlantal cotyles, centrum, and neural arch. The atlantal cotyles comprise broad and anterolaterally expanded projections that bear slightly concave articular surfaces. The centrum surrounds the notochord and also forms the floor of the neural canal. The neural arch is located above the centrum and surrounds the neural canal, being composed of a pair of lateral pedicles connected by a dorsal roof and, in some cases, also a pair of posterior pedicles.

In anterior view, the atlantal cotyles are ventral or ventrolateral to the neural canal. They may be only partially separated by a ventral notch, by a ventral and a dorsal notch, or completely separated from each other by an intercotylar gap that is occupied by an anteromedial part of the centrum. The outline of the neural canal may be circular, elliptical, or rhomboidal, and the canal may be relatively broad or narrow. Within the neural canal, spinal cord supports (medially projecting paired processes on the internal walls of the lateral pedicles) may be distinct or poorly developed (i.e., limited to a faint bone thickening or a groove) or entirely absent. Details of spinal cord supports in caecilian vertebrae are beyond the scope of the current work and will be addressed elsewhere.

In lateral view, a variably developed anterior notch emarginates each lateral pedicle in most but not all caecilians. Heights and lengths of lateral pedicles vary substantially. Immediately above the posterodorsal margin of the lateral pedicles, but anterior to the postzygapophyses, a rounded neural arch notch may be well developed, inconspicuous, or absent depending on the species. A pair of posterior pedicles, usually extending from the posterodorsal margin of the atlantal cotyles, may be distinguished from the main body of the lateral pedicles in some caecilians. The articular surface of the atlantal cotyles may be delimited by a ridge, the cotylar rim, which may be distinct, faint, or entirely absent. When absent, the margins of the articular surface are not very clear. Behind the cotyles, the atlantal centrum is pierced by a pair of spinal nerve foramina. In most modern caecilians, they are barely visible in lateral view, but in stem fossil forms (e.g., *Eocaecilia* and *Rubricacaecilia* Evans & Sigogneau‐Rusell, 2001) and some living species, they are laterally positioned. A posteriorly directed supracotylar protuberance may be present above each spinal nerve foramina and an infracotylar protuberance, a ventrally or laterally directed lip of bone, may be present at the base of the posterolateral margins of the atlantal cotyles. Both protuberances exhibit variation in shape and development among caecilians.

In dorsal view, the anterior margin of the neural arch may be nearly straight, rounded, or pointed, and the posterior margin may be nearly straight, slightly concave, or bear a medial notch. The neural arch roof may be broad and expanded laterally, almost completely covering the atlantal cotyles, or narrower. It may be completely smooth (i.e., lacking any kind of process) or exhibit a distinct but faint medial longitudinal neural spine extending along most of its length. A few caecilians also bear typically paired secondary dorsal crests posteriorly above the postzygapophyses, but these seem quite variable and may be present only on one side of the neural arch. Salamander atlases may have similar structures (e.g., Macaluso et al., 2022) and the atlas of the stem‐caecilian *Rubricacaecilia* has a similarly positioned ‘dorsal tubercle’ that might be homologous with secondary dorsal crests.

In posterior view, the neural canal is surrounded by the neural arch roof dorsally, by the lateral pedicles laterally, and by the posterior pedicles posteroventrally. The neural arch roof is typically depressed medially and may bear an opisthozygosphene, a posteromedial, posteroventrally directed lumenal prominence in the roof of the neural arch occasionally present and variably well‐developed in caecilians. The postzygapophyses are visible at the ventral margins of each side of the neural arch roof with their articular facets oriented ventrally or more obliquely. Lateral and posterior pedicles may be thick or narrow. The spinal nerve foramina are visible posteriorly in some caecilians in which they are posterolaterally directed. The posterior cotyle, the posterior part of the atlantal centrum, bears a concave surface, the notochordal fossa, which may be deep or shallow. The outline of the posterior cotyle is usually oval due to the ventral projection of a posteromedial sagittal process from the centrum or more circular when the process is absent. Two postcotylar fossae are present behind the articular facets of the atlantal cotyles, next to the atlantal centrum, and in some cases, foramina may be present within these fossae.

In ventral view, caecilians exhibit a medially positioned atlantal ventral fossa, a deep or shallow pit, between the atlantal cotyles. When present, a medially positioned hypapophyseal keel may extend anteriorly from the base of the posterosagittal process. The articular facets of the postzygapophyses are better visualized in the ventral view, and their outline shape is quite varied, depending mainly on the presence or absence of the neural arch notches.

### 4.2 Trunk vertebrae

The following description focuses on well‐developed trunk vertebrae as are found at mid‐body. Separate sections provide additional information on the second vertebra and on terminal vertebrae where these differ substantially.

**FIGURE 5:**
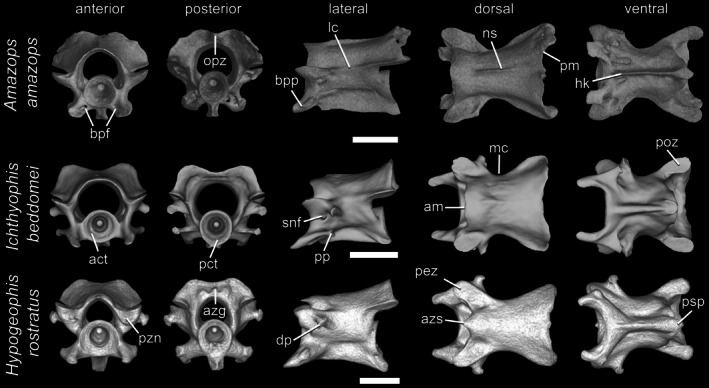
Trunk vertebrae of *Amazops amazops*, *Ichthyophis beddomei*, and *Hypogeophis rostratus*, in anterior, posterior, lateral, dorsal, and ventral views. Scale bars = 1 mm. act, anterior cotyle; am, anterior margin of the neural arch; azg, azygantrum; azs, azygosphene; bpf, basapophyseal foramen; bpp, basapophyseal process; dp, diapophysis; hk, hypapophyseal keel; lc, lateral crest; mc, medial constriction; ns, neural spine; opz, opisthozygosphene; pct, posterior cotyle; pez, prezygapophysis; pm, posterior margin of the neural arch; poz, postzygapophysis; pp, parapophysis; psp, posterosagittal process; pzn, prezygapophyseal notch; snf, spinal nerve foramen.

A well‐formed (non‐rudimentary) trunk vertebra (Figure 5) consists of an amphicoelous centrum and a neural arch. It articulates with the preceding vertebra via its prezygapophyses, anterior cotyle, and, when present, basapophyseal processes. Articulation with the following vertebra occurs via its postzygapophyses, posterior cotyle, and posterosagittal process. Non‐rudimentary trunk vertebrae are rib‐bearing and articulate with the bicapitate ribs via dorsal and ventral transverse processes (= diapophysis and parapophysis, respectively). They form the bulk of the caecilian vertebral column and are readily characterized by bearing a set of distinct anatomical features, which are described in detail below. In contrast, posteriormost trunk vertebrae (referred to here as terminals) are very simple in terms of morphology (i.e., rudimentary) and lack most of these typical features.

The centra of well‐formed caecilian trunk vertebrae usually have a distinctive medial constriction giving them a characteristic hourglass shape. The bony surfaces of their typically subcircular anterior and posterior cotyles form notochordal fossae that are conical, concave, and typically deep hollows. Within the neural canal, the centra have either a rounded dorsal surface or a dorsal keel with or without a medial gap. Medial, longitudinal, hypapophyseal keels separate the ventral grooves on the ventral surface of the centrum and may be robust or more or less narrow and pointed. Posteriorly, the keel merges with the enlarged posteroventrally directed posterosagittal process that projects beyond the posterior cotyle and between the basopophyseal processes of the succeeding vertebra that is so distinctive of well‐developed caecilian trunk vertebrae.

The neural canal, which varies in width within and between species, is bounded by the centra and neural arch, the latter comprising mostly robust, but not high, paired lateral pedicles of variable length and a neural arch roof. Spinal cord supports are usually absent, and when present, typically they are limited to faint paired medially directed protuberances from the internal walls of the lateral pedicles. A spinal nerve foramen may pierce the lateral pedicles in only the anteriormost trunk vertebrae or throughout much of the column, and may be subdivided by a short, transverse bony ridge into two smaller foramina. When present, neural spines are a medial low ridge usually extending along the entire neural arch roof length, but in some cases, they are restricted to its posterodorsal region. They are usually best developed on anteriormost vertebrae, where they may be more obvious in lateral view, but in posterior trunk vertebrae they are limited to faint ridges. The shape of the anterior margin of the neural arch is mostly gently convex in dorsal view but varies across species and with positioning in the column, and they may project more strongly anteriorly and reach the level of the prezygapophyses when an azygosphene is present. The outline of the posterior margins of the neural arch varies from straight to slightly concave in dorsal view and is mostly rounded in posterior view, in some cases, with a posteromedial depression. Posteriorly, a distinct opisthozygosphene may be present medially between the postzygapophyses. Alternatively, in some caecilians, there is an azygantrum, a shallow medial fossa, in the posteroventral surface of the neural arch roof to accommodate the azygosphene of the following vertebra.

Zygapophyses bear cartilages on their articular surfaces, and intervertebral cartilages lie between and mediate the articulation of the cotyles. Paired prezygapophyses bear dorsally oriented articular facets that are not visible in lateral view. A prezygapophyseal notch may be present anteriorly, forming a small shelf below the articular facets. The portions of the neural arch immediately above the prezygapophyses each have a suprazygapophyseal groove, which, in some trunk vertebrae, may be particularly deep and are pierced by a large foramen. A lateral crest (i.e., a ridge of bone) may extend between pre‐ and postzygapophyses. Postzygapophyses are posterior on the neural arch and bear ventral or ventrolaterally facing articular facets that vary in size and shape. They are more or less lateral or dorsolateral and mark the widest point of the neural arch roof. Together with the posterosagittal process, they give the posterior aspect of the vertebrae a somewhat triangular outline.

Well‐developed trunk vertebrae bear paired posterolaterally directed dorsal transverse processes (i.e., diapophyses) and ventral transverse processes (i.e., parapophyses) projecting from the base of the lateral pedicles and the anterior centrum, respectively. In almost all trunk vertebrae, paired basapophyseal processes project anteriorly from the centrum, connected to the ventral transverse processes and ventrolateral to the anterior cotyle. In the postatlantal vertebrae of some forms, the basapophyseal processes may be pierced at their base by small basapophyseal foramina, and a ventral ridge may be present on each basapophyseal process. Transverse processes are better developed in anterior trunk vertebrae, and may be limited to faint protuberances in the posteriormost ones. Transverse and basapophyseal processes give vertebrae a somewhat squarish appearance in anterior view.

When present, many features including the neural spine, transverse processes, hypapophyseal keels, and the posterosagittal processes are usually more pronounced in the anteriormost trunk vertebrae and clearly visible in anterior, but gradually become limited to faint processes or ridges in more posterior vertebrae before disappearing completely.

### 4.3 Second vertebrae

**FIGURE 6:**
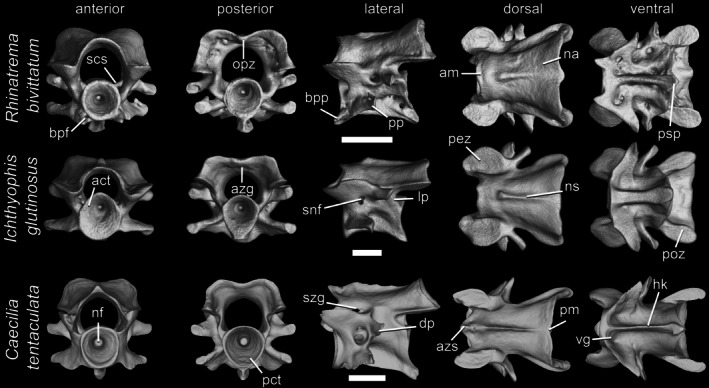
Second vertebrae of *Rhinatrema bivittatum*, *Ichthyophis glutinosus*, and *Caecilia tentaculata*, in anterior, posterior, lateral, dorsal, and ventral views. Scale bars = 1 mm. act, anterior cotyle; am, anterior margin of the neural arch; azg, azygantrum; azs, azygosphene; bpf, basapophyseal foramen; bpp, basapophyseal process; dp, diapophysis; hk, hypapophyseal keel; lp, lateral pedicles; na, neural arch; nf, notochordal fossa; ns, neural spine; opz, opisthozygosphene; pez, prezygapophysis; pm, posterior margin of the neural arch; poz, postzygapophysis; pp, parapophysis; psp, posterosagittal process; scs, spinal cord supports; snf, spinal nerve foramen; szg, suprazygapophyseal groove; vg, ventral groove.

The second (first postatlantal, second nuchal) vertebra (Figure 6) differs from other trunk vertebrae primarily in having a relatively much shorter centrum, with the medial constriction less pronounced or absent. Additionally, well‐developed basapophyseal processes are mostly, though not universally, lacking, and ribs are typically shorter and thicker. When basapophyseal processes are present, their ventral grooves are less conspicuous. Both dorsal and ventral transverse processes are longer in the second vertebrae, and their articular facets are relatively broad. The posterosagittal process is particularly well‐developed in some species, and a well‐developed hypapophyseal keel extends through almost the entire length of the ventral surface of the centrum. The spinal nerve foramen is usually undivided, although in some species it may be subdivided into two smaller foramina. The neural spine usually extends along most of the neural arch roof but may be absent anteriorly when the atlas bears a well‐developed opisthozygosphene.

### 4.4 Terminal vertebrae

**FIGURE 7:**
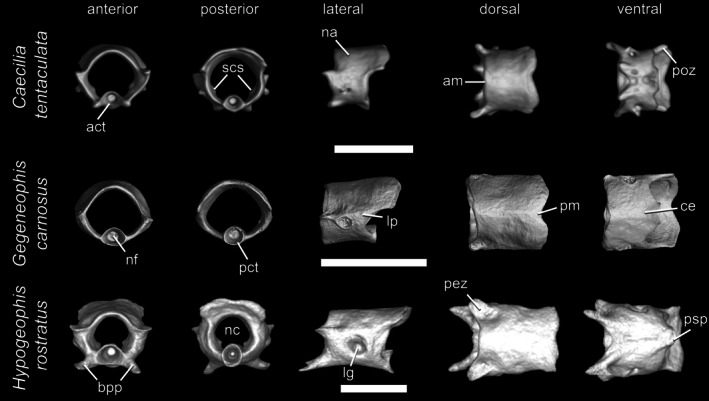
Terminal vertebrae of *Caecilia tentaculata*, *Gegeneophis carnosus*, and *Hypogeophis rostratus*, in anterior, posterior, lateral, dorsal, and ventral views. Scale bars = 1 mm. act, anterior cotyle; am, anterior margin of the neural arch; bpp, basapophyseal process; ce, centrum; lg, lateral groove; lp, lateral pedicles; na, neural arch; nc, neural canal; nf, notochordal fossa; pct, posterior cotyle; pez, prezygapophysis; pm, posterior margin of the neural arch; poz, postzygapophysis; psp; posterosagittal process; scs, spinal cord supports.

Terminal vertebrae (Figure 7) are characterized by shorter and narrower neural canals, shorter centra without a medial constriction, and marked reduction or even complete absence of other major features, including basapophyseal processes, pre‐ and postzygapophyses, hypapophyseal keels, and posterosagittal processes. Anterior margins of the neural arch roof are usually slightly rounded or roughly straight in dorsal view, but rarely an anteromedially projecting azygosphene may be present. Posterior margins of the neural arch roof are usually slightly concave in dorsal view. More rarely, a deep medial notch is present. Dorsal and ventral transverse processes, when present, are usually short and limited to protuberances. In some caecilians, rib‐bearing terminal vertebrae have a fossa (i.e., lateral groove) near the base of the prezygapophyses for articulation with the rib instead of a transverse process. Spinal nerve foramina and basapophyseal foramina are always absent, as are opisthozygosphenes. Spinal cord supports are rarely present. These vertebrae may be well articulated through their typically narrow and short pre‐ and postzygapophyses, but the most terminal elements are often fused to each other or at least appear fused in CT scans. The last terminal vertebrae are rudimentary and limited to incomplete neural arch‐centrum rings. Ribs are usually present in all but the most terminal vertebrae, and in most cases are limited to short rods.

### 4.5 Caudal vertebrae

**FIGURE 8:**
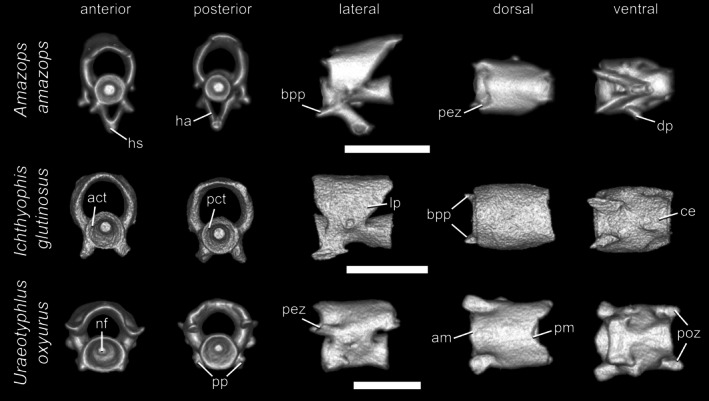
Caudal vertebrae of *Amazops amazops*, *Ichthyophis glutinosus*, and *Uraeotyphlus oxyurus*, in anterior, posterior, lateral, dorsal, and ventral views. Scale bars = 1 mm. act, anterior cotyle; am, anterior margin of the neural arch; ce, centrum; bpp, basapophyseal process; dp, diapophysis; ha, haemal arch; hs, haemal spine; lp, lateral pedicles; nf, notochordal fossa; pct, posterior cotyle; pez, prezygapophysis; pm, posterior margin of the neural arch; poz, postzygapophysis; pp, parapophysis.

True caudal vertebrae (Figure 8) are found exclusively in tailed caecilians. Among extant taxa, only rhinatrematids and ichthyophiids are considered to retain an unambiguous true tail. Caudal vertebrae decrease in size gradually and differ from the posterior trunk vertebrae in having a weak (or even absent) medial constriction, relatively short lateral pedicles of the neural arches, and poorly developed postzygapophyses. They also may differ in the presence of more or less well‐developed haemal arches associated with the basapophyseal processes. Ribs may be present in anterior caudal vertebrae, although like those present in rib‐bearing terminal vertebrae, they are usually rudimentary. Like most trunk vertebrae, caudal ones are also formed by a centrum connected to a neural arch, with the exception of the last 1–3, in which a neural arch may be partially or completely lacking. In some caecilians, the caudal vertebrae may be imbricated.

The neural arch roof is smooth due to the lack of well‐developed neural spines. Most caudal vertebrae have distinct but relatively short prezygapophyses, but they may be completely absent from those located more posteriorly. Similarly, the postzygapophyses are also short and narrow when present, but usually they are missing. The anterior margins of the neural arch may be straight or rounded, but rarely an azygosphene is present. With respect to the posterior margin, both opisthozygosphenes and azygantrum are absent from the caudal vertebrae. Lateral pedicles of the caudal vertebrae exhibit a relatively short length and are not pierced by spinal nerve foramina. Most caecilians lack spinal cord supports in their caudal vertebrae, but they may be retained as poorly developed protuberances in some species. As the diameter of the neural canal decreases in size along the column, it is reduced in caudal vertebrae. Rib‐bearing caudal vertebrae bear diapophyses, although they are limited to faint articular surfaces.

Like the vertebral centra of trunk vertebrae, those from the caudal ones are also amphicoelous and spool‐shaped, bearing rounded anterior and posterior cotyles with deep notochordal fossae. The dorsal surface of the vertebral centra is rounded and relatively smooth due to the absence of dorsal keels. The basapophyseal processes are still present on the anteroventral portion of the vertebral centrum in most of the caudal vertebrae, with the exception of the last ones. However, they project less anteriorly and are not as robust as those of well‐developed trunk vertebrae. Basapophyseal foramina are absent from the basapophyseal processes. In the rib‐bearing caudal vertebrae (usually only the anterior ones), the parapophyses are still present but limited to a faint projection. When present, haemal arches develop at the base of the basapophyseal processes and may be complete (i.e., completely surrounding the haemal canal) or not, being posteroventrally directed and decreasing gradually in size. Alternatively, in some caecilians, they are only vestigial and restricted to faint projections. Even in complete haemal arches, the haemal spine is very short, whereas in incomplete haemal arches, it is absent and the haemal canal remains open ventrally. Unlike the anteriormost postatlantal vertebrae, the ventral surface of the vertebral centrum is usually smooth due to the lack of distinct hypapophyseal keels and posterosagittal processes.

### 4.6 Ribs

**FIGURE 9:**
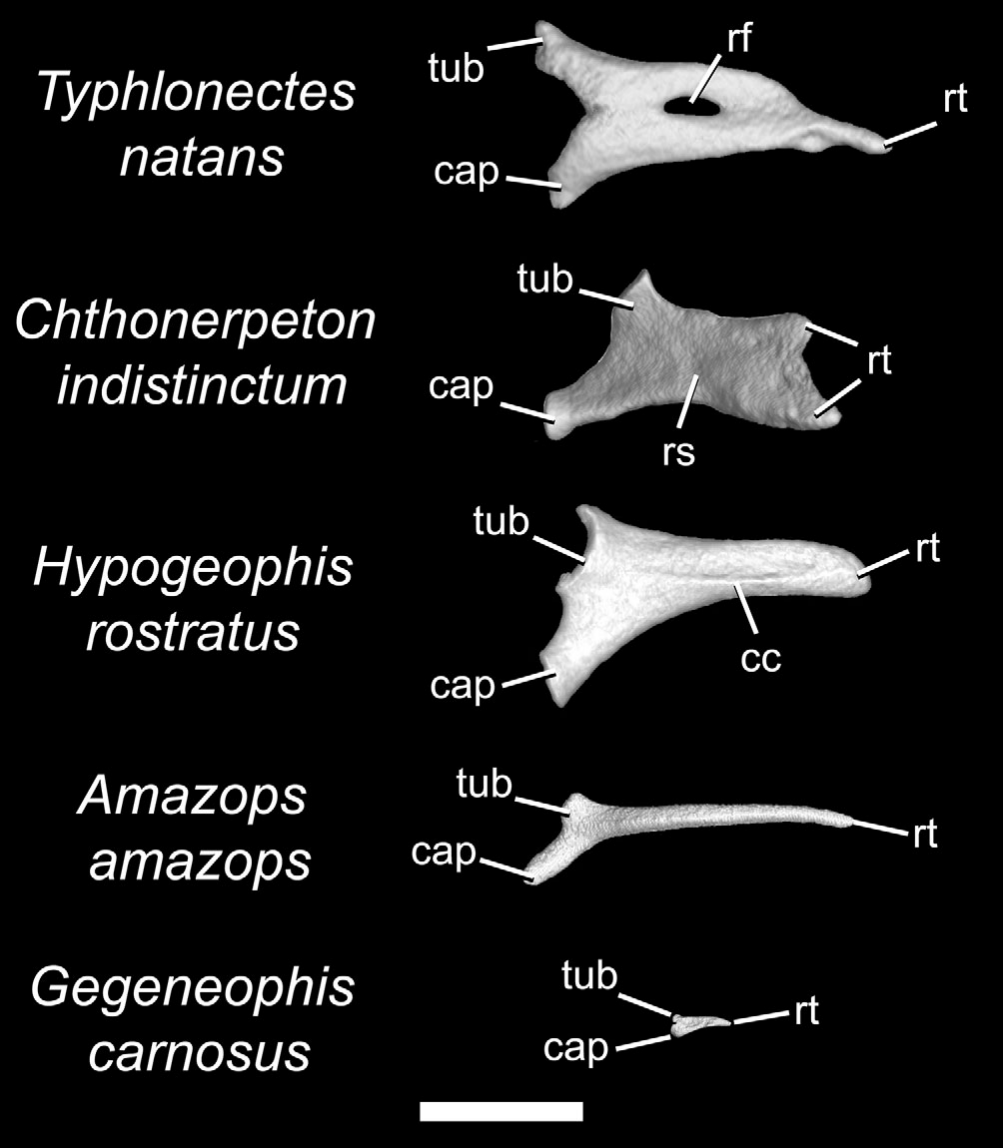
Ribs of *Typhlonectes natans*, *Chthonerpeton indistinctum*, *Hypogeophis rostratus*, *Amazops amazops*, and *Gegeneophis carnosus*, in lateral view. Scale bar = 1 mm. cap, capitulum; cc, costal crest; rf, rib foramen; rs, rib shaft; rt, rib terminus; tub, tuberculum.

Paired short ribs (Figure 9) that do not extend around the coelom articulate with most vertebrae, but their morphological diversity has received little attention. Ribs are absent from the atlas and usually from a few terminal vertebrae. They are relatively simple, and their sizes and shapes vary with position in the column. Well‐developed ribs have an elongate posterolaterally oriented shaft and bicapitate heads, articulating via a short anterodorsally oriented tuberculum with the articular facet of the dorsal transverse process (i.e., diapophysis) and via a longer capitulum with the articular facet of the ventral transverse process (i.e., parapophysis). The anterior surface extending between the capitulum and tuberculum may be straight or concave. The shaft may be fairly straight or have a posterior flexure. It is usually narrow and pointed distally but may be dorsoventrally expanded along its entire length or only anteriorly. Some anteriormost vertebrae, especially the second, may have bifid or forked ribs with distinct upper and lower distal tips. The shaft may bear a short costal crest on its anterior surface, which may be more distinct or limited to a faint ridge. When the costal crest is well‐developed, it may bear a shallow fossa. A foramen may be present in the central portion of the rib shaft. The margins of this foramen may be irregular (i.e., not perfectly rounded). Ribs in the posteriormost region of the column may lack a distinct tuberculum or capitulum, becoming limited to a simple rod‐shaped element or a nubbin.

## 5 DISCUSSION

Despite its long research history, caecilian comparative vertebral osteology is far from complete, with there being no descriptive reports of any vertebrae of the vast majority of the approximately 220 currently known species. There is also substantial variation in anatomical terminology applied to caecilian vertebrae. Our primary aim in this review is to improve the foundations for further research on the caecilian vertebral column. To that end, we provide general descriptive accounts of caecilian vertebrae and ribs that give the main features and highlight the kinds of inter‐and intraspecific variation that we have encountered in our survey of representatives of all 10 currently recognized families of caecilians, and we propose a standardized anatomical nomenclature to allow more precise and less ambiguous use of descriptive terms.

There are clear benefits in using a standardized and non‐redundant terminology when describing anatomical features, especially the avoidance of ambiguities and the facility to recognize homologous structures. Nevertheless, we have tolerated some non‐obfuscatory nomenclatural pluralism. If multiple terms have been exclusively applied to some element, such that these terms are synonyms, then we see no danger in the use of any of the synonyms provided that the relation to synonymous terms is not misunderstood to be anything other than synonymy.

For example, in our terminology, the second vertebra is also the second nuchal vertebra and also the first postatlantal vertebra. These alternative names are unlikely to cause any confusion, and there is no need to propose any standardization that might only limit freedom to employ the most apt usage in any instance of use. In contrast, this vertebra is also sometimes called the axis, but given that caecilians do not have a mammalian‐style atlas‐axis complex (e.g., Wake, 2003) we reject the term axis as potentially mistakenly implying the same function for the second vertebra in caecilians as in mammals or indeed albanerpetids (Fox & Naylor, 1982; Gardner, 2000).

Tolerance of synonyms may be helpful in cases where different names have been applied to homologous structures in different groups or at different hierarchical levels, where any attempt to impose uniformity is unlikely to be uniformly welcomed. Thus, we use both diapophyses and parapophyses (used widely across vertebrates) and their synonyms dorsal and ventral transverse processes (used more commonly across other amphibians, especially salamanders, see Estes, 1981).

### 5.1 Newly recognized structures on caecilian vertebrae: Azygosphene, azygantrum, and opisthozygosphene

We report for the first time the presence of several structures in the vertebral column of caecilians, three of which, the azygosphene, azygantrum, and the opisthozygosphene, are of special interest because of their distinctiveness. The three terms are a clear reference to two structures typically found in the vertebral column of lepidosauromorphs, the zygosphene and the zygantrum (Romer, 1956), which form an additional vertebral articulation involved in the restriction of lateral and dorsoventral bending of the vertebrae (Jurestovsky et al., 2020). However, despite some superficial resemblance, the caecilian counterparts differ significantly from the zygosphene and zygantrum of lepidosauromorphs in several aspects, most notably in being unpaired (azygous) and considerably simpler in terms of morphology, being limited to an anteriorly pointed projection and a shallow pit, respectively. Further, in at least some caecilians, the azygosphene articulates with the opisthozygosphene (see below) of the previous vertebra, and there is no azygantrum. We suggest these medial structures may also play a role in restricting dorsoventral flexing of the regions of the column where they occur.

We name a tuberosity present in the posteromedial margin of the neural arches of some caecilian vertebrae, including the atlas, as the opisthozygosphene, in reference to its occurrence at the opposite end of the neural arch as the lepidosauromorph zygosphene. Some other vertebrates, such as sauropod dinosaurs, have a structure in a similar position traditionally called the hyposphene (e.g., Romer, 1956). Despite the similarity of position, there are three reasons to choose a different terminology for caecilians. Firstly, Gadow (1933) and some followers used “hyposphene” differently for what we refer to here as the posterosagittal process and its use for a different structure would likely cause confusion. Secondly, unlike the opisthozygosphenes of caecilians, archosaur hyposphenes contribute to an accessory intervertebral articulation between trunk vertebrae, along with the hypantrum, and is interpreted as an adaptation for gigantism (Romer, 1956; Stefanic & Nesbitt, 2019). We found no evidence of a hypantrum‐like structure in the vertebral column of caecilians and no correlation with large body size. Thirdly, the distant phylogenetic relationships of the bearers of these traits implies non‐homology.

### 5.2 The terminology of the basapophyseal processes

The presence of a pair of well‐developed, anteriorly directed basapohyseal processes is one of the most distinctive features of caecilian trunk vertebrae. Many different names have been employed for these processes (e.g., parasphenes, processus transversus inferiores, ventral process, hyperapophyses, parapophyses, parapophyseal processes, see Table 2 for a detailed list) over time, and the plethora of alternatives is suboptimal and potentially confusing. In particular, the varied use of the term parapophyses to denote basapohyseal processes is a source of ambiguity.

Here we propose restricting the use of parapophyses to a synonym of the ventral transverse processes. These terms designate only the regions of articulation of the vertebral centrum with the capitula of the ribs, which is continuous with, but distinct from, the basapophyseal processes. The distinction is made particularly clear in the posteriormost vertebrae of some caecilians which exhibit a poorly developed basapophyseal process but completely lack parapophyses and thus do not bear ribs. In terms of function during locomotion, the parapophyses and the basapophyseal processes perform very different roles, with the latter serving for the attachment of intercentral ligaments and basapophyseal muscles (see Naylor & Nussbaum, 1980) related to the articulation of one vertebra with another.

### 5.3 The terminology of the posterosagittal processes and the hypapophyseal keel

Similarly, distinctive of caecilian vertebral morphology is what here we refer to as the posterosagittal process. This is a typically large and robust process that projects posteroventrally from the centrum, below the posterior cotyle, and lies between the basapophyseal process of the following vertebra. This same structure has received many different names over the years (see Table 2), including ones that have quite different meanings in non‐caecilians, and we considered it best to coin a new term for this long‐recognized structure.

By adopting a new terminology, we are actually following the original intention of Gadow (1933) when he proposed the term hyposphene (i.e., that the terminology should reflect functional distinctness), because unlike the hypapophysis of snakes (Romer, 1956), in caecilians this process is also involved in the articulation between the vertebrae. Nevertheless, the term hypapophysis is partially conserved in our terminology because the medial keel present at the ventral surface of the vertebral centra of postatlantal caecilian vertebrae, which is connected to the posterosagittal process, is named hypapophyseal keel. We consider this distinction particularly useful because this keel, like the hypapophysis present in amniotes, serves as an attachment site for the hypaxial muscles.

### 5.4 Comparative anatomy of the vertebral columns among extant lissamphibians

Extant lissamphibians differ greatly in their postcranial osteology, although they still share some morphological features, such as the presence of holospondylous vertebrae (i.e., centrum and neural arch completely fused) and relatively short ribs (Schoch, 2014). The vertebral column is greatly reduced in modern frogs (up to nine presacrals, but usually only eight), although in some salamanders and especially in caecilians, the number of vertebrae is higher (e.g., Duellman & Trueb, 1994). Patterns of regionalization in the vertebral column are better defined in anurans (presacral, sacral, and postsacral regions, see Trueb, 1973) and salamanders (cervical, trunk, sacral, sacrocaudal, and caudal regions recognized, see Worthington & Wake, 1972), but in caecilians, they are poorly defined (see below). Frogs lack a distinct tail when adults, but they exhibit a unique bone, the urostyle, formed by the fusion of the postsacral vertebrae (Ročková & Roček, 2005; Trueb, 1973). In caecilians, tails are very reduced or even absent and rarely bear haemal arches (Wake, 2003), whereas in salamanders, numerous caudal vertebrae are present and the haemal arches are very common.

Salamanders are unique among living lissamphibians in having a well‐developed interglenoid tubercle (i.e., odontoid process) between the atlantal cotyles (Francis, 1934, but see Korneisel et al., 2024). In some anurans, the atlas may be fused to the second presacral vertebra (Trueb, 1973), whereas in salamanders and caecilians, the atlas and the following vertebra always remain separated. Caecilian vertebrae are known to be exclusively amphicoelous, but the shape of the vertebral centra in other lissamphibians may vary; as in some anurans and several salamanders, they may be opisthocoelous (i.e., centrum concave at the posterior end), whereas procoelous (i.e., centrum concave only at the anterior end) vertebrae are rarely present in salamanders but are common to most frogs (Rage et al., 1993; Trueb, 1973).

Vertebral centra tend to be more anteroposteriorly elongated in the trunk vertebrae of caecilians and salamanders when compared to those of anurans. Neural spines are typically found on the neural arches of at least some of the vertebrae of extant lissamphibians. In frogs and salamanders, most vertebrae bear neural spines (Duellman & Trueb, 1994), whereas in caecilians these structures are present only in the anteriormost vertebrae. However, even when they are present, neural spines of lissamphibians are usually limited to low ridges, except in some anurans, in which they may be considerably tall or flattened (Trueb, 1973). The arrangement of the spinal nerve foramina varies in salamanders. In some taxa, they are absent from postatlantal vertebrae, whereas in others, they may be found in most of them (Edwards, 1976). Spinal nerve foramina are common in the atlas and anterior vertebrae of caecilians but are occasionally lacking in those vertebrae located more posteriorly in the column (Wake, 1980). In adult frogs, spinal nerves usually run intervertebrally, but spinal nerve foramina may be present in the urostyle.

Transverse processes are robust and well‐developed in the trunk vertebrae of salamanders and presacral vertebrae of anurans (Duellman & Trueb, 1994). In contrast, in caecilians distinct transverse processes are present only in the anteriormost vertebrae and, even when present, they are relatively short. Ribs in salamanders are usually bicapitate, as in caecilians, but in some taxa, the capitulum and tuberculum may be fused, which constitutes a very useful feature for taxonomy (Larson et al., 2003). Most modern anurans simply lack ribs. However, when present, frog ribs are usually very short, have no distinct projections except uncinate processes (present only in some taxa), and may either be fused to presacral vertebrae or remain separate, being then called free ribs (Blanco & Sanchiz, 2000).

### 5.5 Comparisons between extant and fossil caecilians

Ancient caecilians left few clues of their evolutionary past. Fossils assigned to Gymnophionomorpha (i.e., the group that includes modern caecilians and their limbed ancestors, see Marjanović & Laurin, 2008) are relatively uncommon and usually limited to isolated and fragmented vertebrae (see Santos et al., 2020, and references therein). However, some particularly well‐preserved specimens include still articulated vertebrae, allowing a better characterization of vertebral column morphology. For example, the discovery of the Jurassic *E. micropodia* revealed that elongation of the body was already apparent within the gymnophionomorph lineage since at least the Middle Mesozoic (Jenkins et al., 2007; Jenkins & Walsh, 1993). Based on the little information available about the postcranial osteology of Mesozoic caecilians (especially *E. micropodia*, but also the Triassic *Funcusvermis gilmorei* and the Cretaceous *Rubricaecilia monbaroni*), it is clear that the vertebral morphology of caecilians underwent several changes over time (Evans & Sigogneau‐Russell, 2001; Kligman et al., 2023).

The vertebral morphology of the recently described *F. gilmorei* remains poorly known since only one postatlantal vertebra was found (Kligman et al., 2023). Features that distinguish modern forms from archaic stem caecilians include: absence of an interglenoid tubercle on the atlas (present in *E*. *micropodia*, *R*. *monbaroni*, the condition in *F*. *gilmorei* is unknown), vertebral centrum formed only by the pleurocentrum (*E*. *micropodia* has small intercentra, the conditions of *F*. *gilmorei* and *R*. *monbaroni* are unknown), presence of a hypapophyseal keel (absent in *E*. *micropodia* and only weakly developed in *F*. *gilmorei* and *R*. *monbaroni*), and well‐developed basapophyseal processes (absent in *F*. *gilmorei*, *E*. *micropodia*, and *R*. *monbaroni*).

It is noteworthy that other Mesozoic caecilians already had a morphology more similar to modern forms. Evans et al. (1996) described four trunk vertebrae that have several features typically associated with modern caecilians, including the presence of well‐developed hypapophyseal keels and basapophyseal processes. Regarding Cenozoic fossil caecilians, most of the material found so far comprises broken vertebrae, which are considered basically uninformative with respect to their taxonomic affinities (see Santos et al., 2020, and references therein). However, the vertebrae described by Hecht & LaDuke (1997), despite closely resembling those of extant taxa, stand out for exhibiting an unusually large size.

### 5.6 Representation of postcranial osteological characters in phylogenetic analyses

As noted above, Lescure et al. (1986), Wilkinson (1989), and Wilkinson & Nussbaum (1999) are the only analyses of phylogenetic relationships among living caecilians that have incorporated variation in the morphology and counts of postcranial elements in phylogenetic characters. The scenario is markedly different when one considers analyses that also involve extinct amphibians. Paleontological studies have traditionally paid more attention to the morphology of the postcranial elements but have mainly addressed questions related to the origin of lissamphibians and/or the taxonomic affinities of other extinct amphibian taxa (e.g., Anderson et al., 2008; Marjanović & Laurin, 2019; Ruta & Coates, 2007; Schoch, 2013). With few exceptions (e.g., Kligman et al., 2023; Maddin et al., 2012; Pardo et al., 2017), living caecilian species were rarely included in such analyses, and the few that have been included as Operational Taxonomic Units do not exhibit significant variation in the coding of characters related to postcranial osteology, because these characters were originally intended to capture the variation present in more plesiomorphic forms. For example, some of these characters involve the morphological variation in elements absent in all modern forms (e.g., intercentra, proatlantes, and sacral vertebrae) or that represent plesiomorphic conditions universally retained by all known extant caecilians (e.g., higher vertebral counts and presence of holospondylous vertebrae). As a consequence, the potential utility of morphological variation in the postcranial elements in the phylogenetics of Gymnophiona remains largely untested.

### 5.7 Body segmentation and vertebral column regions in caecilians

Body segmentation is one of the most distinctive features of caecilians, evidenced mainly by the presence of annuli along almost their entire body (e.g., Wilkinson et al., 2011). Caecilians may exhibit primary, secondary, and tertiary annuli (Nussbaum & Wilkinson, 1989), and in the case of primary annuli, there is an approximate 1:1 relationship between them and the vertebrae throughout most of the caecilian body, especially in the trunk region, except for the anterior (in the region of the two nuchal collars there are 3–6 vertebrae) and posteriormost (the last vertebrae are usually very short and often fused to each other) portions of the body (Nussbaum & Wilkinson, 1989).

It is common among vertebrates with elongated bodies that the vertebral column is composed of vertebrae superficially uniform in shape. Moreover, in the case of caecilians, considering that they completely lost both girdles and limbs throughout their evolutionary history, recognizing regions within the vertebral column is especially difficult (Jenkins et al., 2007; Lawson, 1963). Although differences in morphology and measurements of the vertebrae were observed depending on their positioning on the vertebral column (thus supporting the hypothesis of regional differences), both Wake (1980) and Lowie et al. (2022b) did not propose the recognition of any specific vertebral series. In this scenario, external markers (e.g., nuchal collars and the vent) may offer a good alternative to delimit regions in the vertebral column.

The subset of nuchal vertebrae, as proposed by Lescure et al. (1986), includes vertebrae that lie completely or partially within the nuchal collars and is roughly (but not equal) to the concept of cervical vertebrae of Taylor (1977). These anterior most vertebrae (especially V2 and V3) are shorter and bulkier when compared to the posteriormost ones, which is interpreted as an adaptation of the axial skeleton to provide structural support and resist soil reaction forces during burrowing activities (Lowie et al. 2022a). The following trunk vertebrae comprise most of the skeleton, and their number varies greatly intra‐and interspecifically. The posterior trunk vertebrae tend to be more elongate and bear long basapophyseal processes and short dorsal and ventral transverse processes, being considered well‐adapted to provide significant surface area for muscle attachment and facilitate locomotion through the substrate (Lowie et al. 2022a).

In the limbed *E. micropodia*, although the pectoral girdle was preserved, its positioning in relation to the vertebrae could not be determined exactly due to shifts that occurred during fossilization, hampering the identification of a distinct cervical region (Jenkins et al., 2007). However, Jenkins et al. (2007) were able to recognize the sacral and caudal series due to the preservation of the pelvic girdle. Even in modern caecilians, mainly in species with true tails (e.g., rhinatrematids and ichthyophiids), the recognition of a caudal series is at least easier than in those species lacking true tails (e.g., Wilkinson et al., 2021). The body termini of tailless caecilians end abruptly and almost do not extend beyond the cloaca (Wilkinson & Nussbaum, 2006). Even so, the posteriormost vertebrae of such forms resemble those of tailed species in some features (e.g., absence of basapophyseal processes and well‐pronounced hypapophyseal keels), but differ in others (e.g., haemal arches are never present in tailless caecilians).

### 5.8 Relationship between trunk musculature and vertebral morphology in caecilians

Due to the complete absence of limbs and girdles, caecilians rely entirely on their axial musculature to execute their normal locomotion and burrowing (Naylor & Nussbaum, 1980), and the musculature is adapted both to generate the high amount of push force required during head first burrowing and for locomotion within burrows (Summers & O'Reilly, 1997). Caecilians are capable of performing four different types of movements (internal and external concertinas, and lateral and swimming undulations) depending on the kind of substrate or the width of the tunnel they are in (O'Reilly et al., 2000; Summers & O'Reilly, 1997).

Although caecilians closely resemble salamanders in relation to the morphology of the epaxial muscles (formed by *M*. *dorsalis trunci* and *M*. *interspinalis*), they differ substantially in the hypaxial musculature (Naylor & Nussbaum, 1980; Nussbaum & Naylor, 1982). Unlike salamanders, caecilians exhibit basapophyseal muscles and a unique layer of *M*. *subvertebralis* (*M*. *subvertebralis pars ventralis*), which are directly related to their locomotor abilities and inserted on the vertebrae (Naylor & Nussbaum, 1980; Nussbaum & Naylor, 1982). The presence of this set of muscles obviously led to significant changes in the vertebral morphology of the caecilians.

It is worth noting that, unlike burrowing reptiles (i.e., amphisbaenians, snakes, and some other squamates), the vertebral column of caecilians is composed of biconcave (amphicoelous) vertebrae, whereas these reptiles bear ball‐and‐socket (i.e., procoelous) intervertebral joints (Naylor & Nussbaum, 1980). As a result, the articulation between the caecilian vertebrae is formed by a unique arrangement, in which a pair of well‐developed and anteriorly directed basapophyseal processes articulates with the centrum of the anterior vertebra through the intercentral ligaments (Naylor & Nussbaum, 1980). The basapophyseal processes also serve as the insertion points for the basapophyseal muscles, which combined with the system of intercentral ligaments, provide the strengthening of the vertebral column (Naylor & Nussbaum, 1980).

Some members of the family Typhlonectidae, especially the species fully adapted to the aquatic environment, are unable to perform internal concertina locomotion and rely entirely on lateral undulation to move (Summers & O'Reilly, 1997). These differences impact the morphology of their hypaxial muscles (characterized by the presence of complex basapophyseal muscles and reduced *M*. *subvertebralis pars ventralis*) and vertebrae (exhibiting features such as well‐developed hypapophyseal keels and posterosaggital processes, as well as pronounced ventral ridges on the basapophyseal processes) (Nussbaum & Naylor, 1982). Despite the fact that previous studies have considered that the trunk musculature of caecilians is only slightly variable (Nussbaum & Naylor, 1982), unfortunately, the morphological characterization of the muscles of most species is still scarce, hampering the establishment of correlations between vertebral morphology and aspects related to myology.

## 6 CONCLUSIONS

Although vertebrae and ribs are the most abundant elements in the caecilian skeleton, they have been constantly neglected in the study of caecilian osteology. This scarcity of works focused on postcranial osteology, along with inconsistencies in the anatomical terminology of vertebral structures, led to problems related to ambiguity in anatomical definitions and, consequently, in the recognition of homologous structures, hampering studies of comparative anatomy. Both vertebrae and ribs appear to exhibit a considerable morphological variation among the caecilian taxa, but few studies explored their potential as a source of taxonomic data (e.g., Wilkinson & Nussbaum, 1999). We expect that the clarified anatomical definitions proposed in this work, along with the data of the correlated trunk musculature, facilitate the definitions of new character states, which may potentially lead to an improvement in morphology‐based phylogenies of caecilians. In addition, our work will be particularly useful for further paleontological studies, as the bulk of the caecilian fossil record is composed of isolated and damaged vertebrae (e.g., Santos et al., 2020, and references therein), which were traditionally considered poorly informative regarding their taxonomic utility due to the lack of diagnostic features.

## AUTHOR CONTRIBUTIONS

**Rodolfo Otávio Santos:** Writing – original draft; conceptualization; investigation; methodology; visualization; formal analysis. **Mark Wilkinson:** Conceptualization; funding acquisition; methodology; validation; writing – review and editing; data curation; supervision; resources. **Hussam Zaher:** Data curation; supervision; resources; project administration; writing – review and editing; funding acquisition; validation; methodology; conceptualization.

## Supporting information


**Data S1.** Supporting Information.

## Figures and Tables

**FIGURE A1:**
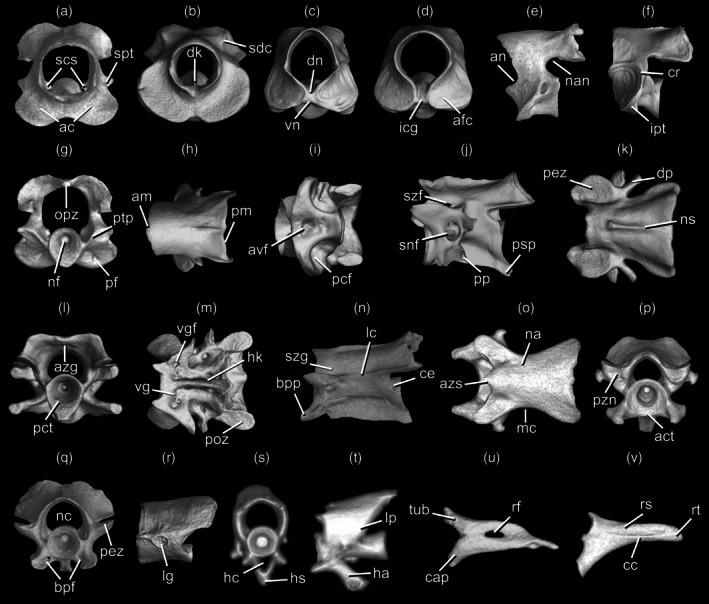
Illustrated glossary containing different elements of the caecilian vertebral column. (a–i) atlases, (j–m) second vertebrae, (n–q) anterior trunk vertebrae, (r) terminal vertebra, (s and t) caudal vertebra, (u and v) ribs. Vertebrae depicted in “a,” “e,” “g,” “m,” “n,” “q,” “s,” and “t” are from *Amazops amazops*, in “b,” “k,” and “l” from *Ichthyophis glutinosus*, “c,” “f,” and “j” from *Caecilia tentaculata*, “d” and “i” from *Siphonops annulatus*, “h” from *Chthonerpeton indistinctum*, “o” and “p” from *Hypogeophis rostratus*, and “r” from *Gegeneophis carnosus*. Rib depicted in “u” is from *Typhlonectes natans*, and in “v” from *H. rostratus*. Figures are not in scale. ac, atlantal cotyles; act, anterior cotyle; afc, articular facets of the atlantal cotyles; am, anterior margin of the neural arch; an, anterior notch; avf, atlantal ventral fossa; azg, azygantrum; azs, azygosphene; bpf, basapophyseal foramen; bpp, basapophyseal process; cap, capitulum; cc, costal crest; ce, centrum; cr, cotylar rim; dk, dorsal keel of the vertebral centrum; dn, dorsal notch; dp, diapophysis; ha, haemal arch; hc, haemal canal; hk, hypapophyseal keel; hs, haemal spine; icg, intercotylar gap, ipt, infracotylar protuberance; lc, lateral crest; lg, lateral groove; lp, lateral pedicles; mc, medial constriction; na, neural arch; nan, neural arch notch; nc, neural canal; nf, notochordal fossa; ns, neural spine; opz, opisthozygosphene; pcf, postcotylar fossa; pct, posterior cotyle; pez, prezygapophysis; pf, postcotylar foramen; pm, posterior margin of the neural arch; poz, postzygapophysis; pp, parapophysis; psp, posterosagittal process; ptp, posterior pedicles; pzn, prezygapophyseal notch; rf, rib foramen; rs, rib shaft; rt, rib terminus; scs, spinal cord supports; sdc, secondary dorsal crests; snf, spinal nerve foramen; spt, supracotylar protuberance; szf, suprazygapophyseal foramina; szg, suprazygapophyseal groove; tub, tuberculum; vg, ventral groove; vgf, ventral groove foramina; vn, ventral notch.

## Data Availability

The datasets generated and/or analyzed during the current study are available from the corresponding author upon reasonable request.

## APPENDIX A

### A.1 Morphological abbreviations

ac: atlantal cotyles; act: anterior cotyle; afc: articular facets of the atlantal cotyles; am: anterior margin of the neural arch; avf: atlantal ventral fossa; azg: azygantrum; azs: azygosphene; bpf: basapophyseal foramen; bpp: basapophyseal process; cap: capitulum; cc: costal crest; ce: centrum; cr: cotylar rim; dk: dorsal keel of the vertebral centrum; dn: dorsal notch; dp: diapophysis; ha: haemal arch; hc: haemal canal; hk: hypapophyseal keel; hs: haemal spine; icg: intercotylar gap, ipt: infracotylar protuberance; lc: lateral crest; lg: lateral groove; lp: lateral pedicles; mc: medial constriction; na: neural arch; nan: neural arch notch; nc: neural canal; nf: notochordal fossa; ns: neural spine; opz: opisthozygosphene; pcf: postcotylar fossa; pct: posterior cotyle; pez: prezygapophysis; pf: postcotylar foramen; pm: posterior margin of the neural arch; poz: postzygapophysis; pp.: parapophysis; psp: posterosagittal process; ptd: posterior pedicles; pzn: prezygapophyseal notch; rf: rib foramen; rs: rib shaft; rt.: rib terminus; scs: spinal cord supports; sdc: secondary dorsal crests; snf: spinal nerve foramen; spt: supracotylar protuberance; szf: suprazygapophyseal foramina; szg: suprazygapophyseal groove; tub: tuberculum; vg: ventral groove; vgf: ventral groove foramina; vn: ventral notch.

Glossary (Figure A1).

Anterior cotyle (act): subcircular anterior articular facet of a postatlantal vertebral centrum bearing a concave notochordal fossa.

Anterior margin of the neural arch (am): the anterior edge of the neural arch roof, which in caecilians may differ in outline primarily due to the presence of structures such as the azygosphene.

Anterior notch of the neural arch (an): weak to robust incisure that may be present on the anterior margin of the lateral pedicles of the atlas.

Articular facets of the atlantal cotyles (afc): slightly concave surfaces of the atlantal cotyles that articulate with the occipital condyles.

Atlantal cotyles (ac): paired, broad, anterolaterally expanded projections of the atlas that bear articular facets.

Atlantal ventral fossa (avf): groove located ventrally between the atlantal cotyles.

Atlas: the first vertebra of the column.

Azygantrum (azg): shallow fossa located medially in the posteroventral (lumenal) surface of the neural arch between the postzygapophyses that accommodates the azygosphene of the succeeding vertebra.

Azygosphene (azs): distinct, anteriorly directed projection from the anterior margin of the neural arch that may articulate with the azygantrum or the opisthozygosphene of the preceding vertebrae.

Basapophyseal foramina (bpf): paired foramina occasionally present in the bases of the basapophyseal processes.

Basapophyseal processes (bpp): caecilian basapophyses form anteriorly directed, paired ventrolateral processes extending from the centrum of most postatlantal vertebrae.

Capitulum (cap): anteriorly directed, ventral ramus of the bicapitate head of a rib that articulates with a ventral transverse process.

Caudal vertebrae: the postcloacal vertebrae of caecilians with postcloacal annuli.

Centrum (ce): azygous, ventromedial structure surrounding the notochord and forming the floor of the neural canal. The centra of the non‐terminal postatlantal vertebrae are amphicoelous, typically spool‐shaped, and usually much longer than the atlantal centrum.

Costal crest (cc): short ridge of bone in the frontal plane on the anterolateral surface of the shaft of a rib.

Cotylar rim (cr): the more or less ridge‐like margins of the articular facets of the atlantal cotyles.

Diapophyses (dp): short paired projections of rib‐bearing vertebrae, each of which articulate with a tuberculum.

Dorsal keel of the vertebral centrum (dk): medially positioned longitudinal keel on the dorsal surface of the vertebral centra (i.e., on the floor of the neural canal) that may be continuous or interrupted by a short medial gap.

Dorsal notch (dn): gap present in the atlantal centrum when the cotyles are separated medially.

Dorsal transverse process: synonym of diapophysis.

Haemal arch (ha): complete or incomplete bony arch formed from ventral outgrowths from the basapophyseal processes of some caudal vertebrae. Haemal arches are usually poorly developed and found only in the anterior postcloacal vertebrae of some true‐tailed species.

Haemal canal (hc): a ventral opening found in some caudal vertebrae bordered by haemal arches laterally and vertebral centra dorsally.

Haemal spine (hs): a typically poorly developed prominence at the apex of a well‐developed (i.e., complete) haemal arch.

Hypapophyseal keel (hk): medial, longitudinal ventral keel, usually extending over most of the ventral surface of the centrum in most of the postatlantal vertebrae. The hypapophyseal keel is connected to the posterosagittal process when this structure is present.

Infracotylar protuberances (ipt): ventrally or laterally directed lip of bone below the atlantal cotyles.

Intercotylar gap (icg): the space between atlantal cotyles when they are separated, formed by the anterior portion of the centrum.

Lateral crest (lc): paired laterally positioned ridges on the lateral pedicles of the neural arch of postatlantal vertebrae connecting the pre and postzygapophyses.

Lateral groove (lg): the fossa located on the lateral pedicles of the neural arch in some terminal vertebrae, serving as an articulation site for ribs.

Lateral pedicles (lp): the lateral walls of the neural arch/canal.

Neural Arch (na): arc‐shaped structure above the vertebral centrum comprising two lateral pedicles connected dorsally by a neural arch roof.

Neural arch notch (nan): a rounded notch present in the atlas and located between the upper portion of the neural arch lateral pedicle and the postzygapophysis.

Neural canal (nc): the channel for the passage of the spinal cord bordered by neural arches and centra.

Neural spine (ns): medial, longitudinal ridge of bone on the neural arch roof.

Notochordal fossa (nf): cavity located on the anterior and/or posterior cotyles of the vertebral centra.

Nuchal vertebrae: postatlantal vertebrae lying completely or partially within the nuchal region.

Opisthozygosphene (opz): moderate posteromedial prominence on the ventral surface of the neural arch roof between the postzygapophyses.

Parapophyses (pp): paired projections of rib‐bearing vertebrae, each of which articulates with a capitulum.

Postatlantal vertebrae: all vertebrae except the atlas. In caecilians without true tails, the term trunk vertebrae is also commonly used to name this set, whereas in tailed species, the set of postatlantal vertebrae comprises both trunk and caudal vertebrae.

Postcotylar foramen (pf): a foramen within a postcotylar fossa.

Postcotylar fossae (pcf): paired fossae posterior to the articular facets of the atlantal cotyles, next to the atlantal centrum.

Posterior cotyle (pct): subcircular posterior articular facet of a vertebral centrum bearing a concave notochordal fossa.

Posterior margin of the neural arch (pm): the posterior edge of the neural arch roof, which in caecilian vertebrae is roughly straight, slightly concave, or has a strong posteromedial notch.

Posterior pedicles (ptp): paired regions forming the posteroventral floors of the neural arch.

Posterosagittal process (psp): azygous, posteroventral projection from the vertebral centrum usually extending beyond the posterior cotyle and between the basapophyseal processes of the succeeding vertebra.

Postzygapophyses (poz): paired posterior processes of the neural arch that articulate with the prezygapophyses of the succeeding vertebra.

Prezygapophyseal notch (pzn): a notch that may be present in the anterior portion of the prezygapophyses of some caecilians, below their articular facets.

Prezygapophyses (pez): paired anterior processes of the neural arch that articulate with the postzygapophyses of the preceding vertebra.

Rib foramen (rf): a small foramen in the central portion of the shaft of a rib.

Rib shaft (rs): this portion comprises almost the entire rib excluding the proximal head region that includes the tuberculum and capitulum.

Rib terminus (rt): the distal portion of the rib. In the anteriormost vertebrae of some caecilians, this portion is forked and has separate dorsal and ventral prongs.

Second vertebra: the vertebra located immediately behind the atlas.

Secondary dorsal crests (sdc): paired posterior, longitudinal ridges on the roof of the neural arch above the region of the postzygapophyses.

Spinal cord supports (scs): small, paired, medially projecting processes of the internal walls of the lateral pedicles immediately above or below spinal nerve foramina.

Spinal nerve foramina (snf): paired neural or neurovascular foramina in the lateral pedicles.

Supracotylar protuberance (spt): a posteriorly directed projection of bone above a spinal nerve foramen.

Suprazygapophyseal foramina (szf): foramina within a suprazygapophyseal groove.

Suprazygapophyseal grooves (szg): paired grooves in the lateral pedicles of the neural arch, just above the articular surfaces of the prezygapophyses.

Terminal vertebrae: vertebrae in the posteriormost region of the vertebral column in species that lack true tails.

Trunk vertebrae: non‐caudal, postatlantal vertebrae.

Tuberculum (tub): anteriorly directed, dorsal ramus of the bicapitate head of a rib that articulates with a dorsal transverse process.

Ventral grooves (vg): paired, longitudinal grooves on the anteroventral surface of the vertebral centrum separated by the hypapophyseal keel.

Ventral groove foramina (vgf): foramina within ventral grooves.

Ventral notch (vn): rounded margin present ventromedially partially separating the atlantal cotyles.

Ventral transverse process: synonym of parapophysis.
