# Supplementary material for: An overview of the postcranial osteology of caecilians (Gymnophiona, Lissamphibia)
Source: Anat Rec (Hoboken). 2025 Jun 6;309(3):674–99. doi: 10.1002/ar.70000 (PMC12882041; doi:10.1002/ar.70000)
Supplement: Supplementary file 1 — Data S1. Supporting Information. [file AR-309-674-s001.docx]

**Supplementary Material**

**An overview of the postcranial osteology of caecilians (Gymnophiona, Lissamphibia)**

Rodolfo Otávio dos Santos, Mark Wilkinson, Hussam Zaher

**CT-Scan parameters**

*Amazops amazops* (Figures 3,5,8, and 9) scan parameters:

Molybdenum target set at 180kV and 200μA; scan data were collected at 2.0 frames per second over 3142 projections in 360°; reconstructed voxel size of 8-10μm.

*Caecilia tentaculata* (Figures 4 and 6) scan parameters:

Molybdenum target set at 60kV and 130μA; scan data were collected at 5 frames per second over 3142 projections in 360°; reconstructed voxel size of 9μm.

*Caecilia tentaculata* (Figure 7) scan parameters:

Molybdenum target set at 110kV and 160μA; scan data were collected at 2.8 frames per second over 3142 projections in 360°; reconstructed voxel size of 12μm.

*Chthonerpeton indistinctum* (Figures 4 and 9) scan parameters:

Molybdenum target set at 60kV and 130μA; scan data were collected at 5.0 frames per second over 3142 projections in 360°; reconstructed voxel size of 8μm.

*Gegeneophis carnosus* (Figures 7 and 9) scan parameters:

Molybdenum target set at 200kV and 200μA; scan data were collected at 2.0 frames per second over 3142 projections in 360°; reconstructed voxel size of 17μm.

*Hypogeophis rostratus* (Figures 5, 7, and 9) scan parameters:

Molybdenum target set at 98kV and 180μA; scan data were collected at 2.0 frames per second over 3142 projections in 360°; reconstructed voxel size of 13μm.

*Ichthyophis* *beddomei* (Figure 5) scan parameters:

Molybdenum target set at 98kV and 190μA; scan data were collected at 2.0 frames per second over 3142 projections in 360°; reconstructed voxel size of 11μm.

*Ichthyophis glutinosus* (Figures 3, 6, and 8) scan parameters:

Molybdenum target set at 95kV and 190μA; scan data were collected at 2.0 frames per second over 3142 projections in 360°; reconstructed voxel size of 14μm.

*Rhinatrema bivittatum* (Figure 6) scan parameters:

Molybdenum target set at 110kV and 155μA; scan data were collected at 2.0 frames per second over 3142 projections in 360°; reconstructed voxel size of 8μm.

*Siphonops annulatus* (Figure 4) scan parameters:

Molybdenum target set at 60kV and 130μA; scan data were collected at 5.0 frames per second over 3142 projections in 360°; reconstructed voxel size of 12μm.

*Typhlonectes compressicauda* (Figure 3) scan parameters:

Molybdenum target set at 150kV and 110μA; scan data were collected at 2.5 frames per second over 3142 projections in 360°; reconstructed voxel size of 15μm.

*Typhlonectes* *natans* (Figure 9) scan parameters:

Molybdenum target set at 115kV and 155μA; scan data were collected at 2.8 frames per second over 3142 projections in 360°; reconstructed voxel size of 14μm.

*Uraeotyphlus oxyurus* (Figure 8) scan parameters:

Molybdenum target set at 98kV and 180μA; scan data were collected at 5.0 frames per second over 3142 projections in 360°; reconstructed voxel size of 11μm.

**List of examined specimens** (figured specimens in bold)

*Amazops amazops* (**USNM 320729**)

*Atretochoana eiselti* (NMW 9144)

*Boulengerula boulengeri* (ZMB13224; BM 1895.11.1.3; MW 883)

*Boulengerula changamwensis* (BM 1892.12.31.45; NMK A5504)

*Boulengerula denhardti* (ZMB 22350)

*Boulengerula fischeri* (BM 2008.606)

*Boulengerula niedeni* (BM 2005.10, BM2005.11)

*Boulengerula spawlsi* (MW 7938)

*Boulengerula taitana* (UMMZ 175129; UMMZ 175138)

*Boulengerula ulugurensis* (MCZ 12388; MCZ 12389; MW 6786)

*Brasilotyphlus braziliensis* (AMNH A51751)

*Caecilia abitague* (UMMZ 89930)

*Caecilia caribea* (MCZ 24520)

*Caecilia dunni* (BM 1916.4.25.31)

*Caecilia gracilis* (MW 5673; MZUSP 159870)

*Caecilia marcusi* (ZSM 79/1982)

*Caecilia nigricans* (BM 1945.9.5.1)

*Caecilia pachynema* (BM 1946.9.5.15)

*Caecilia tentaculata* (**MW 5138**; **MZUSP 136502**)

*Chikila fulleri* (SDB 1304)

*Chthonerpeton braestrupi* (ZMUC.R0234)

*Chthonerpeton indistinctum* (MW 16; **MZUSP 57072**)

*Chthonerpeton viviparum* (BM1947.2.13.86)

*Crotaphatrema bornmuleri* (NMW14859)

*Crotaphatrema lamottei* (MW10203)

*Dermophis gracilior* (BM1901.12.19.137)

*Dermophis mexicanus* (BM1864.1.26.397)

*Epicrionops bicolor* (BM78.1.25.48)

*Epicrionops columbianus* (NRM31365)

*Epicrionops peruvianus* (BM1946.9.5.63)

*Gegeneophis carnosus* (**BM1874.4.29.453**)

*Gegeneophis ramaswamii* (MW 371; MW 679; MW 1098; MW 1449)

*Geotrypetes occidentalis* (BM 1946.9.5.37)

*Geotrypetes seraphini* (MW 5571)

*Gymnopis multiplicata* (BM 1907.10.9.10)

*Gymnopis syntrema* (UMMZ 214091)

*Herpele squalostoma* (MW 4608)

*Hypogeophis alternans* (BM 1907.10.15.144)

*Hypogeophis brevis* (UMMZ 189441; UMMZ221048)

*Hypogeophis larvata* (BM 1987.2112)

*Hypogeophis montanus* (BM 2005.1824)

*Hypogeophis pti* (BMNH 2005.1825)

*Hypogeophis rostratus* (BMNH 1907.10.15.135, **BMNH 1970.285**)

*Hypogeophis sechellensis* (BM 1977.353)

*Ichthyophis asplenius* (MW 3789)

*Ichthyophis beddomei* (MW 489; **MW 2546**)

*Ichthyophis glutinosus* (**MW 1745**)

*Ichthyophis kohtaoensis* (USNM 72293)

*Ichthyophis nigroflavus* (BMHN 1987.2373)

*Ichthyophis orthoplicatus* (MW1725)

*Ichthyophis sikkimensis* (ZMB 50546)

*Ichthyophis supachaii* (ZMH 195)

*Idiocranium russeli* (MW8511)

*Indotyphlus battersbyi* (DEH 11274)

*Luetkenotyphlus brasilliensis* (MCP 2063)

*Luetkenotyphlus insulanus* (MZUSP 945)

*Microcaecilia albiceps* (BMNH 1946.9.5.32)

*Microcaecilia dermatophaga* (MW 5655)

*Microcaecilia nicefori* (AMNH 23387)

*Microcaecilia pricei* (MCZ 36902)

*Microcaecilia supernumeraria* (ZMB 5268)

*Microcaecilia unicolor* (MNHNP 581B)

*Mimosiphonops vermiculatus* (KU 93271; MZUSP 64832; MZUSP 64833)

*Nectocaecilia petersii* (BM 61.9.2.6)

*Oscaecilia bassleri* (BM1956.1.15.84; BM1956.1.15.85)

*Oscaecilia ochrocephala* (BM84.5.9.6; MCZ 4268)

*Oscaecilia zweifeli* (BM 89.9.30.16)

*Potomotyphlus kaupii* (MW 7446)

*Praslinia cooperi* (BM1907.10.15.153; BM1907.10.15.154)

*Rhinatrema bivittatum* (**MW 2395**)

*Rhinatrema nigrum* (ROM 38113)

*Rhinatrema ron* (MZSP60016)

*Schistometopum gregori* (MCZ 20143; MCZ20150; MW 7345)

*Schistometopum thomense* (BM 2000.310; MW 11065)

*Scolecomorphus ulugurensis* (BM 2005.170)

*Scolecomorphus vittatus* (BM 2002.100)

*Siphonops annulatus* (BM 2005.9; MW 2043; **MZUSP 156831**)

*Siphonops paulensis* (BM 1956.1.1.75; SMF 21)

*Siphonops hardyi* (BM 2005.5; BM 2005.8)

*Sylvacaecilia grandisonae* (BM 1969.1589; BM 1969.976)

*Typhlonectes compressicauda* (**MW 7437**)

*Typhlonectes natans* (BM 81.4.9.5; **MW 794**)

*Uraeotyphlus narayani* (BMNH 1940.1.5.1)

*Uraeotyphlus oxyurus* (**MNHNP 4271**)
